# Warming and Nitrogen Addition Induce Asynchronous Taxonomic and Functional Responses and Reorganize Microbial C, N, P Cycling Potential in an Alpine Wetland on the Qinghai–Tibet Plateau

**DOI:** 10.3390/microorganisms14102151

**Published:** 2026-09-23

**Authors:** Wenhua Chang, Weiwei Ma, Jianan Du, Wanpeng He, Niu Zhu

**Affiliations:** 1 College of Forestry, Gansu Agricultural University, Lanzhou 730070, China; changwh@st.gsau.edu.cn (W.C.); 18419149198@163.com (J.D.); 15117289051@163.com (W.H.); 2 College of Prataculture Science, Gansu Agricultural University, Lanzhou 730070, China; 1073324010025@st.gsau.edu.cn; 3 State Key Laboratory of Aridland Crop Science, Gansu Agricultural University, Lanzhou 730070, China

**Keywords:** alpine wetland, warming, nitrogen addition, taxonomic–functional asynchrony, carbon, nitrogen, and phosphorus cycling, shotgun metagenome

## Abstract

Climate warming and atmospheric nitrogen deposition are altering carbon (C), nitrogen (N), and phosphorus (P) cycling in alpine wetlands. However, how these drivers jointly reorganize microbial communities and their biogeochemical cycling potential remains poorly understood, particularly in cold, nutrient-limited alpine wetlands. We conducted a four-year field experiment combining open-top chamber warming and nitrogen addition. Soil physicochemical measurements were integrated with shotgun metagenomic sequencing to assess microbial community structure and the functional potential associated with C, N, and P cycling. The results showed that archaeal and bacterial community composition remained stable following warming and nitrogen addition, whereas fungal composition changed. Functional gene richness was positively correlated with archaeal and bacterial richness but was not correlated with fungal richness, indicating asynchronous responses between taxonomic–functional dimensions. Warming and nitrogen addition did not consistently increase the overall abundance of C, N, and P cycling functional genes. Instead, they induced pathway-specific responses: compensatory shifts among C fixation pathways, decreases in complex C degradation genes, increases in nitrification genes, and decreases in genes associated with P acquisition and homeostatic regulation. Soil moisture and enzyme activities were significantly correlated with C, N, and P cycling genes. These findings highlight domain-specific taxonomic responses and process-specific re-organization of microbial functional potential and emphasize the importance of hydro-thermal constraints when predicting alpine-wetland biogeochemical responses to future warming and increasing N inputs.

## 1. Introduction

Wetlands are transitional zones between aquatic and terrestrial ecosystems and are among the most biogeochemically active ecosystems on earth [1]. Although they occupy only 5–8% of the earth’s terrestrial surface [2], wetlands play disproportionately important roles in carbon (C) and nitrogen (N) storage, biodiversity conservation, and climate regulation [3]. Soil microorganisms represent the most biologically active component of soils and play pivotal roles in nutrient cycling within wetland ecosystems. Soil microbial communities regulate key transformations and interactions among C, N, and phosphorus (P) [4]. These processes include organic C decomposition and fixation, N mineralization, nitrification, and denitrification, as well as inorganic P solubilization and organic P mineralization. In the context of global climate change, soil microorganisms play a critical role in climate feedback through their regulation of nutrient cycling [5,6]. Therefore, understanding how soil microbial communities and their associated C, N, and P cycling functions respond to global climate change is essential for improving predictions of future changes in soil nutrient cycling and ecosystem functioning in wetlands.

Climate warming can directly alter microbial abundance, diversity, and community composition, while indirectly affecting microbial communities and their C, N, and P cycling functions by modifying soil moisture, redox conditions, plant-derived C inputs, and nutrient availability [7,8]. Positive, negative, and even neutral effects of warming on microbially mediated C, N, and P cycling have been reported [9,10]. Microbial responses to warming are often inconsistent across wetland ecosystems because the effects of elevated temperature depend on initial thermal conditions and the availability of moisture and substrates. In cold environments, warming alleviates low-temperature constraints on microbial growth and stimulates extracellular enzymatic activity. Consequently, this accelerates soil organic matter decomposition, C and N mineralization and nutrient release [11]. Enhanced decomposition can subsequently increase the availability of C substrates and mineral nutrients, further altering microbial community composition and metabolic potential. However, soil drying and declining water tables induced by sustained long-term warming, depletion of labile substrates, and microbial thermal acclimation may weaken these stimulatory effects [12]. In addition, as metabolic demands increase with rising environmental temperatures, warming may intensify microbial C and P limitation [13]. Thus, warming may simultaneously restructure microbial communities and alter microbial regulation of soil C, N, and P cycling. Nevertheless, owing to the complexity of the soil microbiome, substantial uncertainties remain regarding its responses to climate warming.

Similarly, anthropogenic activities have resulted in widespread atmospheric N deposition, which regulates microbial communities primarily by altering resource availability and the soil chemical environment [14]. Nitrogen enrichment can alleviate microbial N limitation and increase inorganic N availability, thereby stimulating microbial growth and substrate utilization under N-limited conditions [15]. Recent evidence indicates that long-term nutrient enrichment can alter ammonia-oxidation genes and microbial C metabolic functions, suggesting that nutrient inputs affect not only microbial community structure but also the allocation of microbial metabolic potential [16]. However, prolonged nitrogen enrichment may also modify soil pH, resource stoichiometry, and belowground plant C inputs, thereby reshaping microbial communities and leading to reduced microbial diversity or pronounced shifts in community composition [17]. As N limitation is alleviated, microorganisms may reduce their investment in N acquisition while increasing their demand for C or P [18]. This shift in resource limitation may reallocate microbial investment from N acquisition toward C decomposition or P acquiring functions [19]. For example, long-term N inputs can reduce the ecological demand for biological N fixation, while altering nitrification and denitrification functions. Concurrently, induced relative P limitation promotes organic P mineralization, inorganic P mobilization, and high-affinity P transport. Consequently, the effects of nitrogen addition may extend beyond the N cycle and increasingly influence C and P cycling.

More importantly, climate warming and enhanced N deposition often occur simultaneously in wetland ecosystems, and their combined effects may not be simply additive [20]. When warming increases microbial metabolism and nutrient demand, additional N inputs may alleviate warming-induced increases in N demand, thereby promoting microbial growth and organic matter decomposition [21]. Conversely, once nitrogen limitation is relieved, the predominant limiting factor may shift toward soil moisture or P availability, causing nitrogen addition to weaken or even offset the stimulatory effects of warming [22]. Their combined effects may therefore generate synergistic, antagonistic, or other non-additive responses through shifts in resource limitation. However, how these interacting global-change drivers jointly regulate the coupled microbial functions associated with C, N, and P cycling remains poorly understood.

Bacteria, fungi, and archaea differ substantially in substrate utilization strategies, stress tolerance, and redox niches, and their sensitivities to environmental change may also vary [23]. Consequently, even when overall microbial diversity remains relatively stable under warming and nitrogen addition, these global-change drivers may substantially alter microbial community composition and biogeochemical functions by selectively favoring particular functional groups. Functional redundancy may further decouple taxonomic responses from functional responses [24,25]. Evidence from degraded alpine wetlands has shown that microbial C cycling genes can change markedly even when overall bacterial and fungal diversity remains relatively stable [26], suggesting that the taxonomic and functional dimensions of the microbiome may respond differently to environmental perturbations [27]. Similarly, studies in permafrost peatlands have reported increased abundances of *nifH*, *amoA*, and *mcrA* following nitrogen addition, indicating enhanced potential for N fixation, ammonia oxidation, and methanogenesis, respectively [28]. However, most previous studies have focused on a single microbial group or an individual elemental cycle, with comparatively little attention to the tight interconnections among C, N, and P cycling. Consequently, it remains unclear whether microbial taxonomic and functional dimensions respond synchronously to warming and nitrogen addition. Furthermore, their quantitative impacts on C, N, and P cycling genes are still unclear. This knowledge gap is particularly important in high-altitude and high-latitude ecosystems, where low temperatures, high soil moisture, and strong nutrient constraints render microbially mediated processes highly sensitive to environmental change [29].

The Qinghai–Tibetan Plateau (QTP) has experienced pronounced climate warming in recent decades and is simultaneously being influenced by changing anthropogenic inputs of reactive N [29,30]. Alpine wetlands are widely distributed across the QTP, with alpine wet meadows accounting for approximately half of the total alpine wetland area [31]. Because alpine ecosystems have long developed under cold and relatively nutrient-poor conditions, organic matter decomposition is generally constrained, favoring the accumulation of substantial soil C and nutrient stocks [32,33]. These same environmental constraints, however, also make microbial processes particularly sensitive to changes in temperature, moisture, and nutrient supply. Warming may alleviate low-temperature limitation while simultaneously inducing soil drying, whereas nitrogen addition may relieve N limitation but intensify microbial C or P limitation. Their combined effects may therefore alter microbial resource limitation and reshape C, N, and P cycling functions in alpine wetlands [34]. Consequently, microbial responses to warming and N deposition may generate important feedback to regional soil nutrient storage and elemental cycling [35]. To date, most global change studies on the QTP have focused primarily on plant productivity, soil C fluxes, microbial taxonomic diversity, or individual nutrient cycles [29,36]. However, a systematic understanding of how warming and nitrogen addition jointly restructure microbial communities and microbially mediated C, N, and P cycling functions remains lacking.

To address these knowledge gaps, we conducted a four-year field manipulation experiment involving experimental warming and nitrogen addition in an alpine wet meadow at Gahai on the eastern QTP. By integrating soil physicochemical measurements with metagenomic sequencing, we systematically assessed the individual and interactive effects of warming and nitrogen addition on microbial community composition and the functional potential associated with C, N, and P cycling. Specifically, we first quantified the effects of warming, N addition, and their interaction on microbial taxonomic and functional characteristics. We then determined whether the treatments were associated with uniform or element-specific changes in microbial genes involved in C, N, and P cycling. Finally, we evaluated potential non-additive responses and identified the soil environmental factors most closely associated with functional reorganization. Given that alpine wetlands are chronically constrained by low temperature and N availability, we hypothesized that (1) microbial taxonomic responses would be domain specific, with fungal community composition and richness responding more strongly to warming and nitrogen addition than bacterial and archaeal communities; (2) taxonomic richness and functional gene richness would respond asynchronously to the treatments, while the composition of C, N, and P cycling genes would exhibit process-specific rather than uniform changes; and (3) the combined warming and nitrogen addition treatment would produce non-additive responses, with some functional responses being weaker than the additive expectation because simultaneous alleviation of thermal and N limitation could intensify limitation by soil moisture or P availability. By resolving these taxonomic and functional responses, this study aims to improve mechanistic understanding of microbial feedback to climate warming and N enrichment in alpine wetlands and to provide an empirical basis for incorporating microbial regulation into future biogeochemical models.

## 2. Materials and Methods

### 2.1. Study Site

The experiment was conducted in an alpine wet meadow at the Gahai-Zecha Wetland Station on the eastern Tibetan Plateau (34°16′ N, 102°26′ E, ca. 3450 m a.s.l.) in Gannan Tibetan Autonomous Prefecture, Gansu Province, China (Figure 1). The climate is humid alpine, with a mean annual temperature of 1.2 °C (ranging from −9.1 °C in January to 10.5 °C in July) and mean annual precipitation of 782 mm (with more than 80% falling during the growing season from May to October) [37]. According to the Food and Agriculture Organization (FAO) soil classification system, the soil is classified as a typical swamp meadow soil with a sandy loam texture. The vegetation is dominated by *Kobresia* species, mainly including *Carex tibetica*, *Carex muliensis*, *Thalictrum aquilegiifolium*, *Artemisia sacrorum*, and *Artemisia linearifolia* [38].

### 2.2. Experimental Design

To simulate future climate warming and nitrogen deposition, we established four treatments: control (*CK*), warming (*W*), nitrogen addition (*N*), and warming and nitrogen addition (*WN*), and each treatment included three replicate plots. Each plot covered an area of 2.2 m^2^, and adjacent plots were separated by a buffer distance of more than 5 m to minimize edge effects (Figure S1). Passive hexagonal open-top chambers (OTCs) were installed to simulate warming [39]. The approach has been widely applied in warming experiments on the QTP [40]. Each OTC consisted of six trapezoidal transparent acrylic panels assembled into a hexagonal frustum. The chambers were 0.61 m in height, with bottom and top side lengths of 0.91 and 0.63 m, respectively. Beginning in 2021, the bottom edges of the OTCs were tightly inserted into the soil to maintain year-round passive warming.

Atmospheric nitrogen deposition in the Gahai wetland ecosystem is currently estimated to be about 10 kg N ha^−1^ yr^−1^, which may increase by 2 times in the future. Therefore, starting from 2021, a nitrogen addition rate of 20 kg N ha^−1^ yr^−1^ was adopted to simulate future regional atmospheric nitrogen deposition levels [41]. Ammonium nitrate (NH_4_NO_3_) was used as the N source because it supplies both NH_4_^+^ and NO_3_^−^, the principal inorganic forms of atmospheric N deposition [42]. Specifically, 4.14 g of NH_4_NO_3_ was dissolved in 2 L of water and sprayed evenly onto the nitrogen addition subplots on 15 May, 15 July, and 15 October each year. The plots without nitrogen addition received the same volume of water (2 L) to exclude potential effects caused by differences in water input.

### 2.3. Soil and Plant Sampling

Soil and plant samples were collected in mid-August 2024, at the peak of the growing season. Three soil cores (5 cm dia.) were randomly collected from the 0–10 cm soil layer in each plot and homogenized to form one composite sample. In total, 12 composite soil samples (4 treatments × 3 replicates) were collected, transported on ice in insulated coolers, and delivered to the laboratory. All field-moist soil samples were passed through a 2 mm sieve to remove roots, organic debris, and stones. Each composite soil sample was divided into three subsamples for subsequent analyses. The first subsample was immediately stored at −80 °C for high-throughput sequencing and shotgun metagenomic analyses. The second subsample was stored at 4 °C for measurements of soil enzyme activities and labile nutrient pools. The third subsample was air-dried at room temperature to measure other soil physicochemical properties and nutrient concentrations.

The soil properties examined in this study were pH, bulk density (BD), soil water content (SWC), soil temperature (TEM), soil organic carbon (SOC), total nitrogen (TN), total phosphorus (TP), carbon-to-nitrogen ratio (C:N), carbon-to-phosphorus ratio (C:P), nitrogen-to-phosphorus ratio (N:P), dissolved organic carbon (DOC), dissolved organic nitrogen (DON), dissolved organic carbon-to-nitrogen ratio (DOC:N), ammonium nitrogen (NH_4_^+^-N), nitrate nitrogen (NO_3_^−^-N), urease (URE), sucrase (SUR), phosphatase (PHO), nitrate reductase (NR), and nitrite reductase (NIR). The soil property examination method is detailed in the Supplementary Materials (Text S1).

At the same time, all aboveground vegetation within a 0.5 m × 0.5 m quadrat in each plot was clipped at the soil surface and collected in sealed plastic bags to determine aboveground biomass. Belowground plant biomass was sampled from the 0–10 cm soil layer using a root corer (8 cm dia.). The root samples were transported to the laboratory, carefully washed with water, and separated from stones and other debris. Both aboveground and belowground plant samples were oven-dried at 65 °C to a constant mass and then weighed. Biomass was expressed as dry mass per unit ground area.

### 2.4. Metagenomic Sequencing and Analyses

Total soil DNA was extracted from 0.5 g of homogenized soil using the Fast DNA^®^ SPIN Kit for Soil (MP Biomedicals, Irvine, CA, USA) according to the manufacturer’s instructions. The concentration of the extracted DNA was measured using a Qubit 4.0 fluorometer (Invitrogen, Carlsbad, CA, USA). Soil DNA purity was assessed using a NanoDrop 2000 spectrophotometer (Thermo Fisher Scientific, Wilmington, DE, USA). Approximately 1 μg of qualified DNA from each sample was used for shotgun metagenomic library preparation. Shotgun metagenomic libraries were constructed and sequenced on an Illumina NovaSeq 6000 platform (Illumina Inc., San Diego, CA, USA) by Majorbio Biopharm Technology Co., Ltd. (Shanghai, China). An average of approximately 10 Gb of raw sequence data was generated per sample, and the sequencing statistics are provided in Table S1.

Raw paired-end reads were quality-filtered using Trimmomatic (v0.36). Adapter sequences and low-quality terminal bases were removed, reads containing >10% ambiguous nucleotides were discarded, and reads shorter than 75 bp after trimming were excluded. Reads matching the human reference genome were removed as potential contaminant sequences using Bowtie2 (v2.4.5). The resulting high-quality reads were assembled de novo using MEGAHIT (v1.1.2). Open reading frames (ORFs) were predicted from the assembled contigs using MetaProdigal (v2.6.3) and translated into amino acid sequences. Predicted genes from all samples were clustered using CD-HIT (v4.6) at 95% sequence identity and 90% alignment coverage, with the longest sequence in each cluster retained as the representative sequence to construct a non-redundant gene catalog. High-quality reads from each sample were mapped back to the non-redundant gene catalog, and gene abundances were quantified as transcripts per million (TPM) using Salmon (v0.7.2). Taxonomic annotation was performed by comparing representative protein sequences against the National Center for Biotechnology Information (NCBI) non-redundant protein database using BLASTP (v2.2.1). The abundance of each taxon was calculated by summing the TPM values of genes assigned to that taxon.

KEGG Orthology (KO) annotations were assigned using KofamScan (v1.3.0) based on profile hidden Markov models. To characterize microbial biogeochemical functions, representative protein sequences were additionally annotated against CCycDB (https://github.com/ccycdb/ccycdb.github.io/ accessed on 8 September 2026), NCycDB (https://github.com/qichao1984/NCyc accessed on 8 September 2026), and PCycDB (https://github.com/ZengJiaxiong/Phosphorus-cycling-database accessed on 8 September 2026) to identify genes involved in soil C, N, and P cycling, respectively. The abundance of each functional gene family was calculated by summing the TPM values of genes assigned to that family, and the abundance of each metabolic process was calculated as the sum of the abundances of its constituent gene families. Detailed information on the identified C, N, and P cycling genes is provided in Tables S2–S4.

### 2.5. Data Analysis

All statistical analyses were performed in R software version 4.5.1 [43]. The normality and homogeneity of variance of the data were assessed using the Shapiro–Wilk test and Levene’s test, respectively. When necessary, data were log10-transformed to meet the assumptions of normality and homoscedasticity. One-way analysis of variance (ANOVA) was used to examine differences in soil physicochemical properties, plant variables, and soil microbial diversity; the carbon, nitrogen and phosphorus cycling process; and functional gene abundance among treatments. When significant treatment effects were detected, least significant difference (LSD) tests were performed using the “agricolae” (v1.3-7) package at a significance level of α = 0.05. Linear mixed-effects models were used to evaluate the effects of different treatments on soil microbial diversity; the carbon, nitrogen and phosphorus cycling process; and functional gene abundance. Warming and nitrogen addition and their potential interactions were included as fixed effects, whereas plots were included as random effects [44].

Treatment responses of taxonomic and functional gene richness relative to *CK* were quantified using the natural log response ratio (lnRR). Linear regression analysis was used to clarify the relationship between taxon and functional richness. We further quantified the individual, main, and interactive effects of warming and nitrogen addition on taxon and functional richness and on carbon, nitrogen, and phosphorus cycling processes. The main effect of warming, the main effect of nitrogen addition, and the interaction effect of warming × nitrogen addition was further expressed using Hedges’ g [45], and forest plots were plotted with 95% confidence intervals. A detailed formula was provided (Text S2). Spearman correlation analysis was used to evaluate the relationship between environmental factors and the abundance of functional genes for carbon, nitrogen, and phosphorus cycling. The *p* value was adjusted for multiple comparisons using the Benjamini–Hochberg (BH) method. The False Discovery Rate (FDR) was set at the 0.05 significance level. The Mantel test was used to demonstrate the overall correlation between C, N, and P cycle functional gene matrices and environmental variables, whereas Bray–Curtis distance was used for functional gene matrices and Euclidean distance was used for standardized environmental variables.

## 3. Results

### 3.1. Effects of Soil and Plant Factors

The treatments significantly altered plant biomass, soil properties, nutrient availability, and enzyme activities (Table S5). Compared with *CK*, only *WN* significantly increased AGB, whereas *W* resulted in the highest BGB and TB; *N* also increased TB, while BGB under *WN* did not differ from *CK*. Both W and WN increased soil temperature and decreased SWC, whereas W alone reduced soil pH, while *N* and *WN* increased BD. SOC was lower under *W* than under *N* and *WN* (*p* < 0.05), whereas TN was higher under *WN* than under *W*. Both *W* and *WN* markedly decreased TP and consequently increased C:P and N:P, while C:N was lowest under *WN*. Neither DOC nor DOC:N differed significantly among treatments, although *W* increased DON. All three treatments increased NH_4_^+^-N relative to *CK*, whereas NO_3_^−^-N was highest under *W* and *N*, intermediate under *WN*, and lowest under *CK*. Urease and sucrase activities generally declined under the treatments, phosphatase activity was highest under *N*, nitrate reductase activity was higher under *W* and *N* than under *CK*, and nitrite reductase activity decreased in the order *CK* > *N* > *WN* > *W* (*p* < 0.05).

### 3.2. Species Composition of Microbial Community

The classification annotation results of the non-redundant gene catalog identified 19 phylum and 502 genus archaea, 172 phylum and 17,712 genus bacterial, and 7 phylum and 395 genus fungal communities (Figure S2). Across treatments, archaeal and bacterial communities retained broadly similar compositions, whereas fungal communities exhibited greater compositional variation (Figure 2 and Figure S3). At the phylum level, Thermoproteota overwhelmingly dominated the archaeal community, with TA-21 as the predominant genus across all treatments. Bacterial communities were mainly composed of *Acidobacteriota*, *Pseudomonadota*, and *Methylomirabilota*; *W* and *WN* showed an apparent decrease in *Pseudomonadota* accompanied by an increase in *Acidobacteriota* relative to *CK*, while genus-level composition varied only modestly. In contrast, *Mucoromycota* generally increased under *W*, *N*, and particularly *WN*, whereas *Ascomycota* decreased relative to *CK*. Fungal genera also showed greater variation among treatments. *Rhizophagus* was more prominent under *W* and *WN*, whereas *Entrophospora* and *Funneliformis* showed greater contributions under *N* and *WN*.

### 3.3. Response of Soil Microbial Taxonomy and Functional Gene Richness

Richness responses of archaea, bacteria, fungi, and functional genes differed markedly among treatments (Figure 3). Compared with *CK*, archaeal richness increased by 13.0%, 15.4%, and 16.6% under *W*, *N*, and *WN*, respectively, whereas functional gene richness increased by 4.0%, 4.7%, and 5.5%, respectively. Both archaeal and functional gene richness were significantly higher under *WN* than under *CK* (*p* < 0.05). In contrast, fungal richness reached its lowest level under *WN*, while bacterial richness did not differ significantly among treatments. Relative to *CK*, *WN* increased archaeal and functional gene richness by 16.5% and 5.5%, respectively; decreased fungal richness by 43.7%; and increased bacterial richness by only approximately 3.0%. In addition, the ACE and Chao1 indices of both archaea and bacteria were significantly higher under *WN* than under *CK* (*p* < 0.05), whereas no significant differences were detected among the other treatments. The Shannon and Simpson indices showed no significant differences among the four treatments for either archaeal or bacterial communities (Figures S4–S6).

Factorial analysis revealed pronounced group-specific responses of richness metrics to *W* and *N* (Figure 4; Table S6). Warming exerted significant negative effects on fungal richness and functional gene richness (*p* < 0.01), whereas *N* significantly increased archaeal richness and functional gene richness (*p* < 0.05). The positive effect of *N* on archaeal richness was significant (*p* < 0.05). *W* and *N* showed antagonistic interactions for archaeal, bacterial, and fungal richness, although none of these interactions were statistically significant. In contrast, a significant antagonistic interaction was detected for functional gene richness.

The ΔlnRR values between functional gene and bacterial richness were close to zero under *W*, *N*, and *WN*, indicating similar magnitudes of relative change between the two metrics (Figure S7). By comparison, ΔlnRR values between functional gene and archaeal richness were −0.08, −0.10, and −0.10 under *W*, *N*, and *WN*, respectively, indicating that the increase in archaeal richness was greater than that in functional gene richness. In contrast, ΔlnRR values between functional gene and fungal richness were 0.46, 0.21, and 0.63 under *W*, *N*, and *WN*, respectively, reflecting an overall decline in fungal richness despite increases in functional gene richness. The divergence between functional gene and fungal richness was greatest under *WN* and reached marginal significance (Figure 5). Correlation analysis further showed that functional gene richness was significantly and positively correlated with archaeal richness (*p* < 0.01) and bacterial richness (*p* < 0.01). In contrast, functional gene richness was negatively, but not significantly, correlated with fungal richness.

### 3.4. C, N, and P Cycling Pathways and Functional Gene Composition of Soil Microorganisms

A total of 120 C cycling functional genes were annotated across treatments, exceeding those involved in P (68 genes) and N cycling (53 genes) (Figure 6; Tables S2–S4). Carbon-cycling genes were mainly associated with C fixation and C degradation. C fixation genes were assigned to six pathways: the 3-hydroxypropionate (3-HP) bicycle, 3-hydroxypropionate-4-hydroxybutyrate (3HP/4HB) cycle, Calvin–Benson–Bassham (CBB) cycle, dicarboxylate-4-hydroxybutyrate (DC/4HB) cycle, Wood–Ljungdahl (WL) (WL) pathway, and reductive tricarboxylic acid (rTCA) cycle. C degradation genes were mainly associated with the degradation of starch, hemicellulose, cellulose, chitin, pectin, and lignin (Figure 7). Overall, C degradation genes were more abundant than C fixation genes, with lignin degradation dominating the degradation pool and the CBB and DC/4HB pathways dominating C fixation (Figure S8). Total C cycling gene abundance decreased by 10.9%, 7.3%, and 10.1% under *W*, *N*, and *WN*, respectively, relative to *CK* (*p* > 0.05). Despite this overall decline, several C fixation genes showed pathway-specific increases: *aclA* increased by 359.1% and 235.3% under *W* and *WN* (*p* < 0.05), respectively, whereas *gnd* increased by 8.2% under *WN*. In contrast, warming significantly reduced several genes associated with the 3-hydroxypropionate and 3HP/4HB pathways. Responses were more pronounced for C degradation, with *W* and *WN* significantly reducing hemicellulose and chitin degradation genes (*p* < 0.05). Factorial analysis further showed significant negative effects of warming on lignin, hemicellulose, chitin, and pectindegradation genes (*p* < 0.05), accompanied by marked declines in representative genes such as *treS*, *xylD*, *nagG*, *celB*, and *yesW* (Figure 6; Table S7).

N cycling genes were mainly involved in organic nitrogen degradation and synthesis, denitrification, nitrification, nitrogen fixation, assimilatory nitrate reduction, and dissimilatory nitrate reduction (Figure 7). Organic nitrogen degradation and synthesis accounted for 49.1–51.7% of total nitrogen cycling gene abundance, followed by denitrification (28.1–30.4%), whereas nitrification and nitrogen fixation genes were comparatively less abundant (Figure S9). Total N cycling gene abundance decreased by 7.8%, 4.5%, and 6.8% under *W*, *N*, and *WN*, respectively. In contrast to this overall decline, nitrification gene abundance increased significantly by 84.0%, 62.9%, and 46.7%, respectively (*p* < 0.05), largely driven by pronounced increases in *amoC_B* and *nxrA*. By comparison, N fixation genes significantly declined by 20.0%, 4.5%, and 15.7% under *W*, *N*, and *WN* (*p* < 0.05), respectively, while genes associated with organic N metabolism and dissimilatory nitrate reduction also tended to decrease. The overall abundance of denitrification genes remained comparatively stable.

P cycling genes were primarily assigned to inorganic phosphorus transformation, organic phosphorus metabolism, and phosphorus transport systems (Figure 7). Inorganic phosphorus-related genes contributed the largest proportion (52.9–54.4%), followed by organic phosphorus metabolism (25.0–26.4%) and P transport genes (19.3–21.7%; Figure S10). Total P cycling gene abundance decreased by 12.2%, 7.8%, and 12.3% under *W*, *N*, and *WN*, respectively. Genes involved in inorganic P transformation and organic P metabolism generally declined across treatments, with reductions of 9.6–14.6% and 10.5–16.6%, respectively, and significant differences detected under *N* and *WN* (*p* < 0.05). In contrast, P transport genes remained relatively stable, changing by only −1.2%, +0.7%, and −3.3% under *W*, *N*, and *WN*, respectively.

### 3.5. Factors Affecting C, N and P Cycling and Functional Genes of Soil Microbial Communities

Spearman correlation analysis revealed strong associations of soil moisture, nutrient stoichiometry, and enzyme activities with C, N, and P cycling functional genes (Figure 8). For C cycling, SWC and NIR were positively correlated with several C degradation genes. SWC was significantly positively correlated with *treS*, *glnA*, *yesW*, and *xylD*, while NIR was positively correlated with *treS*, *glnA*, *mcd*, *mch*, *fumC*, and *xylD* (*p* < 0.01). In contrast, *aclA* was strongly negatively correlated with both SWC and NIR (*p* < 0.001) but positively correlated with NH_4_^+^-N. N cycling genes were primarily associated with SWC, NH_4_^+^-N, NIR, and soil C and N availability. The nitrification-related genes *amoC_B* and *nxrA* were strongly negatively correlated with SWC (*p* < 0.001) and negatively correlated with NIR, but positively correlated with NH_4_^+^-N. Similar relationships were observed for *hao*. In contrast, *glnA*, *ureC*, and the DNRA-related gene *nirB* were positively correlated with SWC and/or NIR (*p* < 0.05). P cycling genes were mainly associated with SWC, NIR, TP, and soil C and N pools. The phosphonate metabolism gene *phnI* and phosphate-starvation response genes *phoP* and *phoU* were positively correlated with SWC and NIR (*p* < 0.01). The phosphate transport gene *pstC* was positively correlated with TP and PHO, whereas *ugpC* was positively correlated with SWC, NIR, and TP. The polyphosphate synthesis gene *ppk* was also positively associated with SWC. In contrast, *opd* and *pepM* were negatively correlated with SWC and/or NIR. In addition, *phnJ* showed a strong negative correlation with NO_3_^−^-N, whereas *pafA* was positively correlated with pH, TN, and SOC.

Mantel tests further demonstrated significant associations between the composition of C, N, and P cycling genes and multiple soil properties (Figure 9). In particular, C, N, and P cycling gene profiles were significantly associated with SOC, TN, and TP, respectively. C cycling genes were additionally associated with soil C and N related properties, whereas N cycling genes were significantly related to DON, NO_3_^−^-N, and NH_4_^+^-N. SWC and several enzyme activities, including URE, SUC, PHO, NIR, and NR, were also significantly associated with one or more elemental cycling gene sets. Collectively, these results indicate that variation in microbial C, N, and P cycling functional genes was closely associated with soil moisture, substrate availability, elemental stoichiometry, and enzyme activities.

## 4. Discussion

### 4.1. Effects of Warming and Nitrogen Addition on Soil Microbial Community Structure

After four years of experimental treatments, warming and nitrogen addition did not induce uniform changes in soil microbial communities in the alpine wetland. Archaeal and bacterial communities retained broadly similar dominant compositions across treatments, whereas the fungal community exhibited substantially greater compositional variation (Figure 2). These domain-specific responses partially support Hypothesis 1, which predicted that fungi would respond more strongly to warming and N enrichment than prokaryotic communities. Such contrasting sensitivities are ecologically plausible because archaea, bacteria, and fungi differ markedly in their physiological tolerances, resource-acquisition strategies, growth rates, and dependence on plant-derived substrates. Recent alpine-meadow experiments have likewise demonstrated that warming and N enrichment can affect bacterial and fungal diversity asymmetrically and that their responses depend strongly on plant productivity, soil moisture, nutrient availability, and treatment intensity [46,47]. Thus, the absence of a uniform microbial response in our study is consistent with an increasing body of evidence showing that global-change effects on soil microbiomes are strongly domain and context dependent.

The highly consistent dominance structure of the archaeal community across treatments suggests that this community may be subject to strong environmental filtering. *Thermoproteota* includes archaeal lineages associated with ammonia oxidation, some of which are well adapted to low temperatures and low substrate concentrations and may contribute to nitrification in high-elevation soils [48,49]. In the present study, W induced declines in soil moisture and pH, and together with the increase in available nitrogen caused by *N* (Table S3), jointly altered the thermal, substrate, and redox environments experienced by archaea. Nevertheless, these environmental changes did not significantly alter the dominance of *Thermoproteota* [50]. Notably, archaeal richness was higher under the *WN* treatment, whereas the relative abundance of the dominant phylum and community evenness changed only marginally. This pattern suggests that the combined treatment may have primarily promoted the occurrence or persistence of low-abundance archaeal lineages rather than causing replacement of the dominant taxa.

The bacterial community also showed relatively limited compositional change. The compositional stability may reflect a relatively high resistance of alpine wetland bacterial communities to short-term environmental perturbations, but may also indicate a temporal lag in community responses. Previous studies have shown that bacterial communities in alpine meadows changed only slightly after three years of warming [46] but underwent pronounced restructuring after six years of warming [51]. Long-term warming experiments in the Arctic further indicate that detectable shifts in bacterial community composition may sometimes require more than a decade to emerge [52]. Warming decreased soil pH and altered inorganic N availability in our study (Table S3). Soil pH is an important determinant of bacterial community composition [53]. However, these environmental changes may not have been sufficient to reorganize the dominant bacterial assemblage during the four-year experimental period. We also found that nitrogen addition had little effect on overall bacterial community composition or on the relative abundance of the dominant bacterial phyla. One plausible explanation is that nitrogen addition did not significantly alter soil pH in our experiment [53,54]. Although numerous studies have demonstrated that nitrogen addition can play an important role in regulating soil bacterial community structure [55], responses of wetland bacterial communities to N enrichment are highly variable across ecosystems [56].

In contrast, the fungal community was the microbial component most responsive to warming and nitrogen addition in this alpine wetland. Warming altered overall fungal community structure and was accompanied by a decrease in the relative abundance of *Ascomycota* and an increase in *Mucoromycota* (Figure 2). Moreover, the response of *Ascomycota* under the combined *WN* treatment was not fully consistent with that observed under warming alone, indicating that fungal responses to warming and nitrogen addition may depend on the treatment combination rather than simply reflect additive effects. The weaker-than-additive response under WN supported Hypothesis 3. However, some short-term experiments have reported no significant effects of warming or nitrogen addition on fungal community composition [23,57]. These contrasting responses may reflect differences in experimental duration, background soil moisture, plant community composition, and nutrient limitation status [58]. Under warmer and nitrogen-enriched conditions, enhanced soil organic matter mineralization and changes in root exudation can increase the pool of labile C compounds [59], while reducing microbial reliance on relatively recalcitrant organic C substrates [60]. Therefore, warming and nitrogen-induced shifts in rhizosphere C inputs, substrate quality, and inorganic nutrient availability [61] may have restructured resource competition among fungal taxa.

### 4.2. Asynchronous Responses of Microbial Taxonomic and Functional Gene Richness to Warming and Nitrogen Addition

Notably, relative to *CK*, archaeal richness increased under the *W*, *N*, and *WN* treatments, with the largest increase observed under *WN*. This pattern suggests that elevated temperature and enhanced N availability may jointly broaden the ecological niches of certain archaeal taxa. Soil in alpine wetlands is chronically constrained by both low temperatures and limited nutrient availability. Warming may therefore create more favorable conditions for a broader range of archaeal taxa by alleviating thermal limitation, accelerating organic matter mineralization, and increasing the supply of inorganic nitrogen. Nitrogen addition may further expand the niches of ammonia-oxidizing archaea and other archaeal groups capable of utilizing N-containing substrates. Evidence from global wetlands likewise indicates that temperature and soil C:N stoichiometry are important determinants of archaeal diversity [62]. Given that the archaeal community remained strongly dominated by a few major lineages, particularly *Thermoproteota*, the observed increase in richness likely reflects the emergence or persistence of low-abundance archaeal taxa rather than a comparable restructuring of the dominant archaeal assemblage.

In contrast, bacterial richness differed little among treatments. Neither *W*, *N*, nor their interaction significantly affected bacterial richness, indicating relatively high resistance of this taxonomic metric to the experimental treatments. The result was consistent with findings from some long-term warming experiments in alpine meadows [63]. Bacterial communities generally exhibit high initial richness and broad niche differentiation, and opposing responses of different bacterial taxa to changes in temperature, SWC, pH, and nutrient availability may offset one another through taxonomic replacement, thereby maintaining relatively stable overall richness [64]. In addition, nitrogen addition did not significantly decrease pH (Table S3), which may have weakened the filtering effect of N enrichment on bacterial taxa through soil acidification. A study conducted in alpine swamp meadows in an arid region similarly found that nitrogen addition induced changes in taxonomic composition that were not necessarily accompanied by corresponding changes in richness [65]. Global meta-analyses have also shown that the average effect of nitrogen addition on soil microbial richness is generally weak and that both its magnitude and direction are regulated by ecosystem type, nitrogen addition rate, experimental duration, and initial soil pH [56]. Thus, the relative stability of bacterial richness does not imply an absence of bacterial responses to the treatments, but may instead reflect taxonomic turnover among bacterial groups together with a high degree of taxonomic redundancy.

*W*, *N*, and *WN* all reduced fungal richness, with the strongest negative response occurring under *WN*, indicating that fungi were more susceptible to the compound environmental filtering imposed by simultaneous warming and nutrient enrichment. Warming not only directly alters the soil thermal environment but can also indirectly constrain the niches of some fungal taxa by reducing soil moisture, modifying pH and nutrient stoichiometry, and altering plant productivity and belowground C inputs. Soil moisture may be a particularly important regulator of fungal responses in alpine wetlands. Under sufficiently moist conditions, warming can stimulate soil microbial activity, whereas under drought or alternating wet–dry conditions, warming effects may become negative [66]. Reduced soil moisture can also decrease the diffusion and availability of organic substrates, thereby filtering out fungal taxa that are particularly sensitive to desiccation. Warming-induced soil drying may therefore represent an important mechanism underlying the decline in fungal richness. This mechanism may be especially relevant in the moisture-rich but drying-sensitive alpine wetlands of the QTP, where warming-induced declines in soil moisture may exert a stronger control on fungal richness than temperature increases per se. In addition, a warming experiment in coastal wetlands found that fungal communities could be more strongly affected than prokaryotic communities [67], consistent with our observation that fungal richness declined while bacterial richness remained comparatively stable. OTCs may also alter irradiance, wind speed, humidity, snow accumulation and evaporative conditions. These chamber-induced microclimatic changes can influence plant growth and soil biota independently of, or interactively with, temperature. Thus, although OTCs are widely used for alpine warming experiments, the responses observed here cannot be attributed exclusively to temperature.

Interestingly, the fungal response observed here differs from some previous studies reporting increased fungal diversity under warming or nitrogen addition [42,47]. Such discrepancies may largely arise from differences in ecosystem moisture regimes, initial nutrient limitation, and nitrogen addition intensity. The nitrogen addition rate used in the present study was relatively low (20 kg N ha^−1^ yr^−1^) and may have primarily alleviated N limitation without inducing substantial soil acidification or N saturation. The nitrogen did not reduce soil pH but increased aboveground biomass and was accompanied by a tendency toward lower soil moisture, suggesting that the classical soil-acidification mechanism was unlikely to be the main driver of the decline in fungal richness. A more plausible explanation is that enhanced plant growth intensified competition for water and non-N nutrients while simultaneously altering rhizosphere C inputs and substrate composition. Under the *WN* treatment, plant and microbial demands for water, P, or available C may have increased concurrently, shifting the system from predominantly N limitation toward multiple-resource co-limitation and thereby strengthening environmental filtering of fungal taxa.

Overall, taxonomic and functional gene richness did not respond synchronously to warming and nitrogen addition (Figure 5). The clearest directional mismatch occurred between fungal richness, which declined, and functional gene richness, which increased. We therefore interpret this pattern as domain-specific taxonomic–functional asynchrony, supporting Hypothesis 2. This decoupling may arise from microbial functional redundancy, differences among taxonomic groups in their sensitivity to environmental filtering, and the uneven distribution of functional genes across microbial lineages. When environmentally sensitive taxa decline, other taxa carrying similar functional genes may maintain or even expand the potential functional gene pool. Differences in the sensitivity of taxonomic and functional dimensions to environmental change can consequently produce mismatches in both the direction and magnitude of taxonomic restructuring and functional responses [68]. Long-term warming studies have likewise shown that microbial functional gene composition can undergo pronounced reorganization even in the absence of significant shifts in bacterial taxonomic composition, suggesting that functional attributes may be more sensitive to environmental change than taxonomic structure [64]. Similar taxonomic–functional asynchronous responses have also been observed across global wetlands [62]. Therefore, warming and nitrogen addition do not simply increase or decrease overall microbial diversity; rather, they reconfigure the relative contributions of taxonomic and functional diversity through distinct environmental filtering processes. This finding implies that bacterial, fungal, or archaeal taxonomic richness alone may be insufficient to predict changes in the biogeochemical functioning of alpine wetlands under future warming and increasing N deposition.

### 4.3. Effects of Warming and Nitrogen Addition on Microbial Functional Genes Involved in Soil C, N, and P Cycling

Our results showed that sustained warming and nitrogen addition did not consistently increase the abundance of microbial functional genes involved in C, N, and P cycling in the alpine wetland, but instead induced pronounced element-specific and process-specific reorganization (Figure 7). For C cycling, the total abundance and relative contribution of C fixation genes remained generally stable across treatments, whereas the internal composition of individual fixation pathways changed. Warming increased the abundance of *aclA* while decreasing the abundances of *mch*, *mcd*, and *fumC*. This pattern suggests that the apparent stability of overall C fixation potential may result from compensatory changes among different fixation pathways rather than from uniform resistance across all pathways. Compared with C fixation, functions associated with the degradation of complex organic C were more sensitive to warming. Warming significantly decreased the abundance of genes involved in lignin, hemicellulose, chitin, and pectin degradation, which contrasts with some previous studies reporting increased abundances of C degradation genes under warming [11]. Long-term grassland experiments have also shown that warming effects on C degradation functions can be strongly season dependent, occurring mainly during colder periods and becoming weaker during the warmer growing season [69]. These contrasting responses indicate that increasing temperature does not necessarily enhance the degradation of complex organic C, because its effects are jointly constrained by soil moisture, sampling season, substrate availability, and the composition of dominant decomposers. The lower abundance of genes associated with hemicellulose and chitin degradation suggests a change in community genetic potential rather than a decline in measured decomposition rates. Gene abundance did not necessarily synchronize with transcription, enzyme activity, or substrate turnover. Thus, even if the representational abundance of some degradation genes in the metagenome decreases, warming may accelerate some actual decomposition processes through kinetic effects. In our study, SWC was significantly positively correlated with *treS*, *xylD*, *celB*, *nagG*, *yesW*, and several lignin-degradation genes, while Mantel tests also showed a significant association between C cycling functional composition and SWC [12]. These relationships suggest that warming-induced declines in soil moisture may offset the direct stimulatory effects of higher temperature on enzymatic reactions. Reduced soil moisture can restrict the diffusion of soluble substrates and decrease between extracellular enzymes and their substrates. It may also suppress hydrolytic enzyme activity and alter soil redox microsites, thereby constraining microorganisms involved in the degradation of complex organic matter. The prominent association of SWC with microbial functional genes deserves particular attention because alpine wetlands are intrinsically sensitive to hydrological change. Microbial communities in these ecosystems develop under relatively high soil moisture and frequently oxygen-limited conditions; consequently, a reduction in water availability represents more than simple physiological drought stress. Reduced SWC may restrict the diffusion of soluble organic substrates and extracellular enzymes, thereby limiting microbial access to complex C substrates even when higher temperature would otherwise accelerate biochemical reactions.

In contrast to C cycling, the most prominent response of the N cycle was an enhancement in nitrification-related functions. The abundance of nitrification genes was higher under the *W*, *N*, and *WN* treatments than under *CK*, mainly owing to increases in *amoC_B* and *nxrA*. The former is involved in ammonia oxidation, whereas the latter participates in nitrite oxidation. Nitrification genes were positively correlated with NH_4_^+^-N but significantly negatively correlated with SWC, suggesting that substrate availability and soil moisture jointly regulate nitrifying microorganisms. By alleviating low-temperature limitation and reducing soil moisture, warming may increase soil aeration and oxygen availability, thereby favoring aerobic nitrifiers, whereas nitrogen addition directly increases substrate availability for nitrification. These mechanisms may explain the increased abundance of nitrification genes under the individual *W* and N treatments. However, multifactor climate manipulation experiments have shown that drought can exert stronger effects on nitrifying microbial communities than warming or elevated CO_2_. Drought can alter the relative contributions of ammonia-oxidizing archaea, ammonia-oxidizing bacteria, and nitrite-oxidizing bacteria [70]. This is consistent with the strong negative relationship between nitrification genes and SWC observed in our study, suggesting that warming effects may be mediated partly through changes in soil moisture and redox conditions rather than being driven solely by increasing temperature. In contrast, partial drying of moist wetland soils may increase oxygen penetration and soil aeration, creating conditions more favorable for aerobic N transformations such as nitrification. The simultaneous decrease in SWC and increase in nitrification-related genes under warming is consistent with this possibility. Thus, some of the functional reorganizations attributed to warming may have arisen from warming-induced drying and associated changes in soil aeration and resource diffusion rather than from higher temperature alone. In alpine wetlands characterized by low temperature, high soil moisture, and strong diffusion limitation, warming-induced improvements in soil aeration may favor nitrification to a greater extent than in comparatively dry grasslands [71]. More importantly, although warming and nitrogen addition individually enhanced nitrification-related genes, the observed increase under *WN* was substantially lower than the additive expectation. When warming and nitrogen addition occur simultaneously, soil drying, insufficient available C, intensified P limitation, or rapid depletion of NH_4_^+^ may prevent nitrification potential from continuing to increase linearly. In contrast to enhanced nitrification, warming decreased the abundances of *ureC*, *glnA*, and *nirB*, suggesting that organic N hydrolysis and nitrate-to-NH_4_^+^ retention pathways may be suppressed.

Warming significantly decreased the total abundance of P cycling genes as well as the abundances of *phoR*, *phoB*, *phoP*, *phoU*, *pstC*, *ugpC*, *ppk*, and *ppx*. These genes collectively participate in sensing external P status, phosphate uptake, organic P transport, and polyphosphate storage and mobilization. Their concurrent declines therefore suggest that warming may weaken the genetic potential for P acquisition and homeostasis [72]. Enhanced plant growth under warming may increase plant P demand and intensify competition for P between plants and microorganisms. Furthermore, soil drying may reduce P diffusion and mobility P in the soil solution. The positive correlations of SWC with *phnI*, *phoP*, *phoU*, *phoR*, *pstC*, *ugpC*, and *ppk* are also consistent with the interpretation that adequate moisture availability helps sustain microbial P acquisition potential [13]. Drying can modify pore-water connectivity, solute and substrate diffusion, oxygen penetration, and soil redox conditions, thereby simultaneously altering microbial access to C, N, and P resources and the energetic constraints on different metabolic pathways. At the same time, *opd* and *pepM* showed increasing trends despite declines in most P regulatory and transport genes, suggesting that microbial P use strategies may shift from broad phosphate uptake, regulation, and polyphosphate turnover toward a smaller number of organic P or phosphonate utilization pathways [10]. Thus, changes in P cycling are more likely to represent a reconfiguration of strategies for utilizing different P sources rather than a uniform loss of all P-cycling functions.

Overall, after four years of experimental warming and nitrogen addition, microbial functional potential in this alpine wetland showed process-specific reorganization, with a greater relative representation of nitrification-related genes and lower representation of genes associated with complex C degradation and P acquisition. These patterns suggest a potential shift in the balance among microbial C, N, and P cycling strategies rather than a uniform enhancement in or suppression of microbial biogeochemical functions. Several limitations should, however, be considered when interpreting these results. First, soil metagenomes were sampled only once after four years of treatment; therefore, the observed differences represent endpoint treatment-associated patterns rather than temporal trajectories. Second, metagenomic gene abundance reflects genomic potential and does not necessarily correspond to gene expression, enzyme activity, or realized biogeochemical process rates. Third, the OTC treatment simultaneously increased soil temperature and reduced soil moisture, making it impossible to separate direct thermal effects from indirect effects of warming-induced drying and other chamber-related microclimatic changes. Future studies should integrate metatranscriptomics, key enzyme activities, stable-isotope tracing, and direct measurements of C, N, and P process rates to determine whether changes in functional gene abundance translate into realized ecosystem processes. Integrating taxonomic composition, functional gene inventories, gene expression, enzyme activities, and elemental cycling fluxes will help improve predictions of alpine wetland feedback to the combined effects of future warming and increasing N deposition.

## 5. Conclusions

Four years of warming and N addition produced strongly domain- and process-specific microbial responses in this alpine wetland. Fungal communities were more responsive than archaeal and bacterial communities, while taxonomic richness and functional gene richness did not respond synchronously. Metagenomic functional potential was reorganized among C, N, and P cycling processes rather than uniformly enhanced or suppressed, with particularly strong contrasts among complex C degradation, nitrification, and P-acquisition functions. The close association between these functional patterns and soil moisture further suggests that warming-induced hydrological changes may be an important component of microbial responses in alpine wetlands. Because metagenomic abundance represents functional potential rather than realized activity, future work integrating metatranscriptomics, enzyme measurements, isotope tracing, and direct process-rate measurements will be required to determine how these genomic shifts translate into ecosystem biogeochemical fluxes.

## Figures and Tables

**Figure 1:**
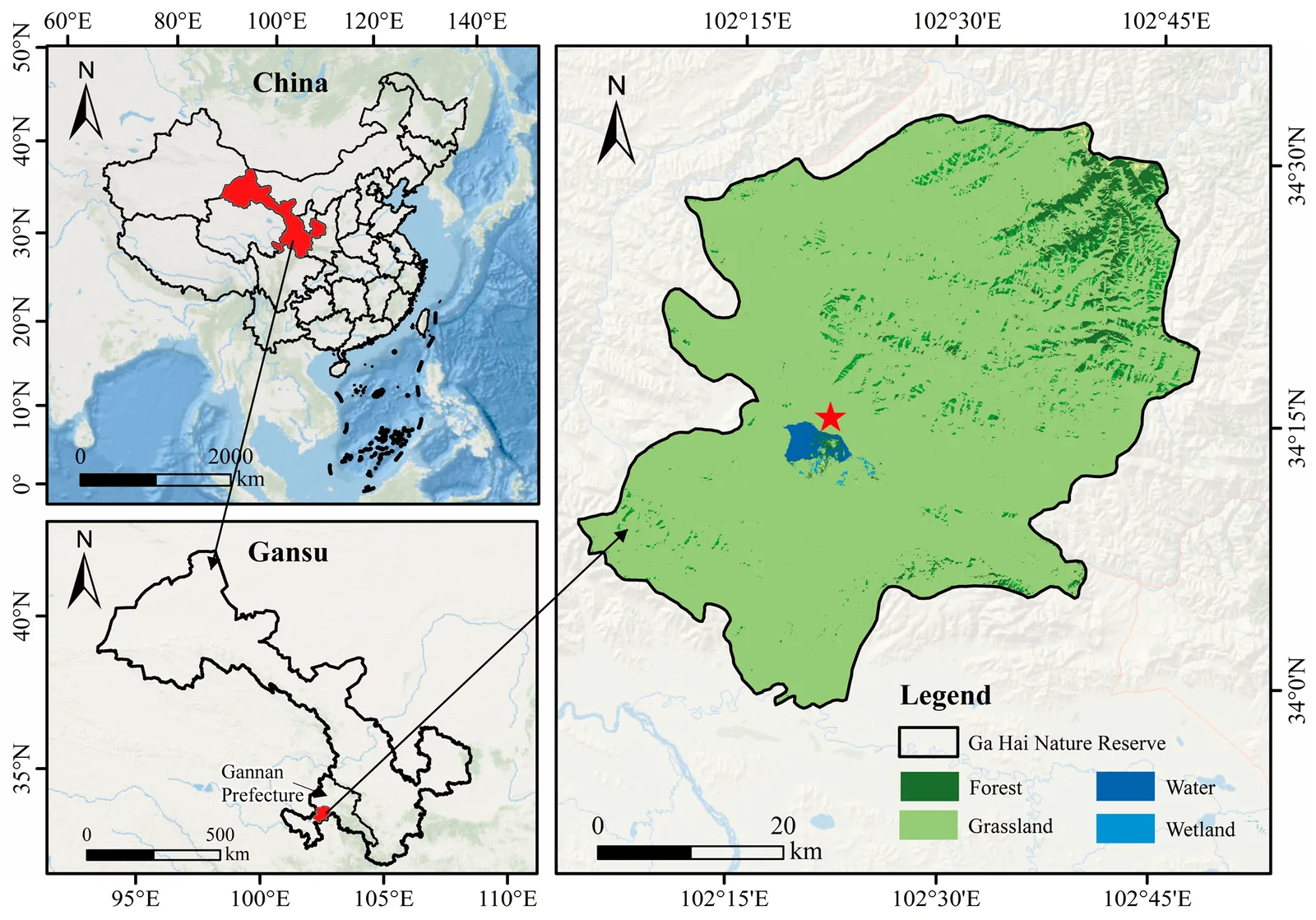
The location of the study area.

**Figure 2:**
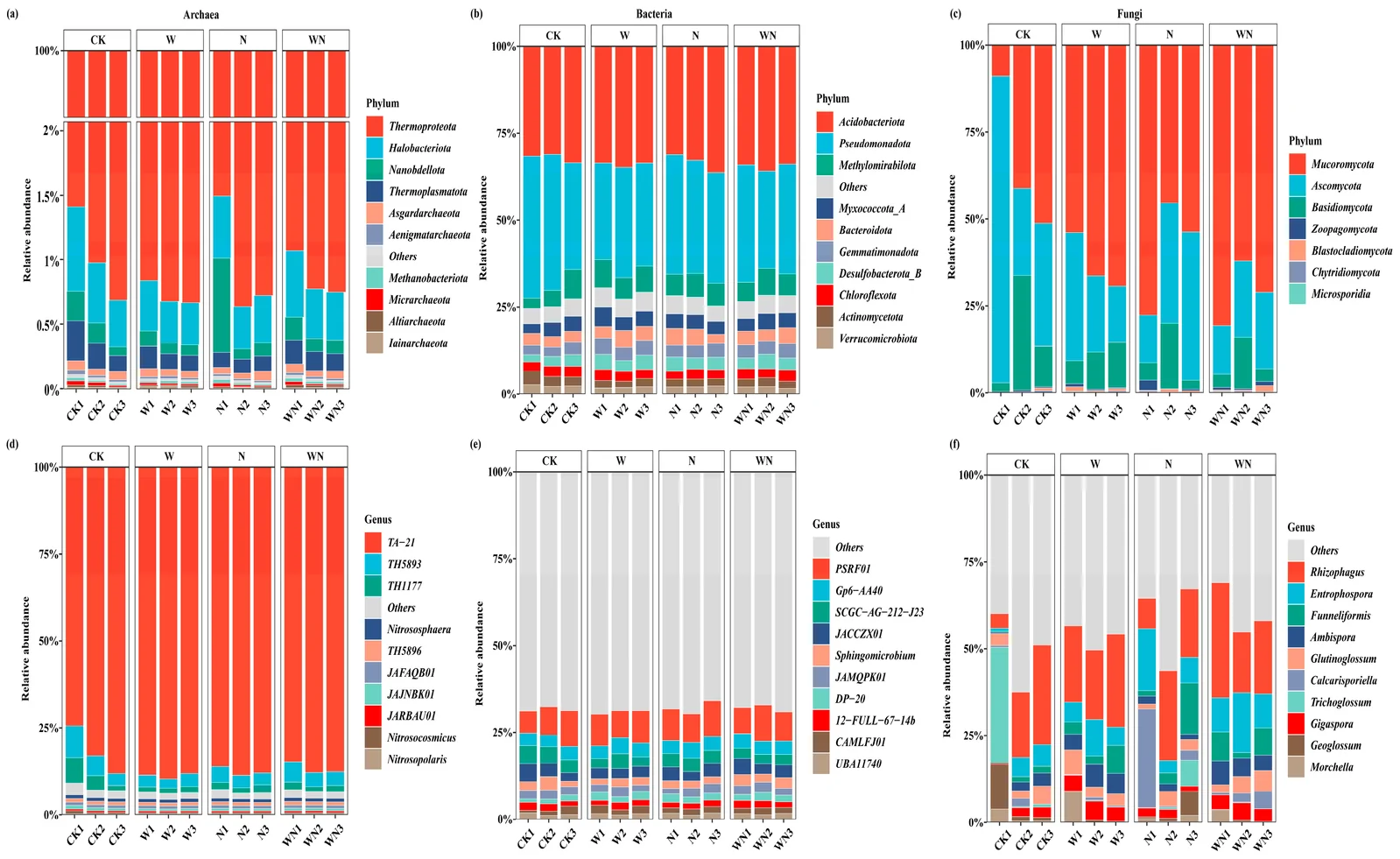
Soil microbial community composition under warming and nitrogen addition. Relative abundances of the 10 most abundant taxa in archaeal, bacterial, and fungal communities at the phylum (**a**–**c**) and genus (**d**–**f**) levels. *CK*, control; *W*, warming; *N*, nitrogen addition; *WN*, combined warming and nitrogen addition. Taxa outside the 10 most abundant groups were pooled as Others, and each stacked bar represents the relative abundance of taxa within an individual sample.

**Figure 3:**
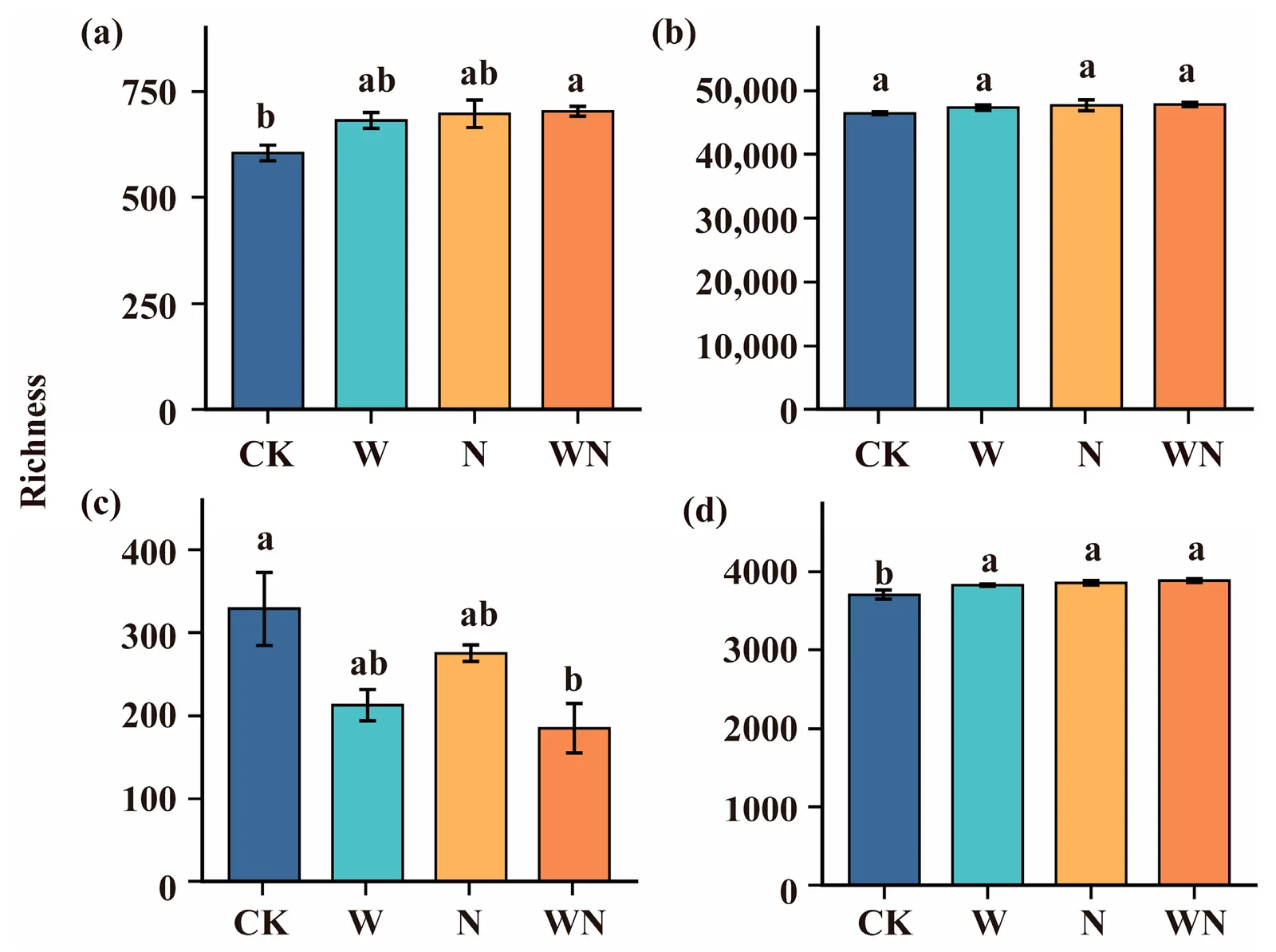
Taxonomic richness of soil archaeal (**a**), bacterial (**b**), and fungal (**c**) communities and functional gene richness (**d**) under warming and N-addition treatments. *CK*, control treatment; *W*, warming treatment; *N*, nitrogen addition treatment; *WN*, warming nitrogen addition treatment. Different lowercase letters indicate significant difference between treatments (*p* < 0.05).

**Figure 4:**
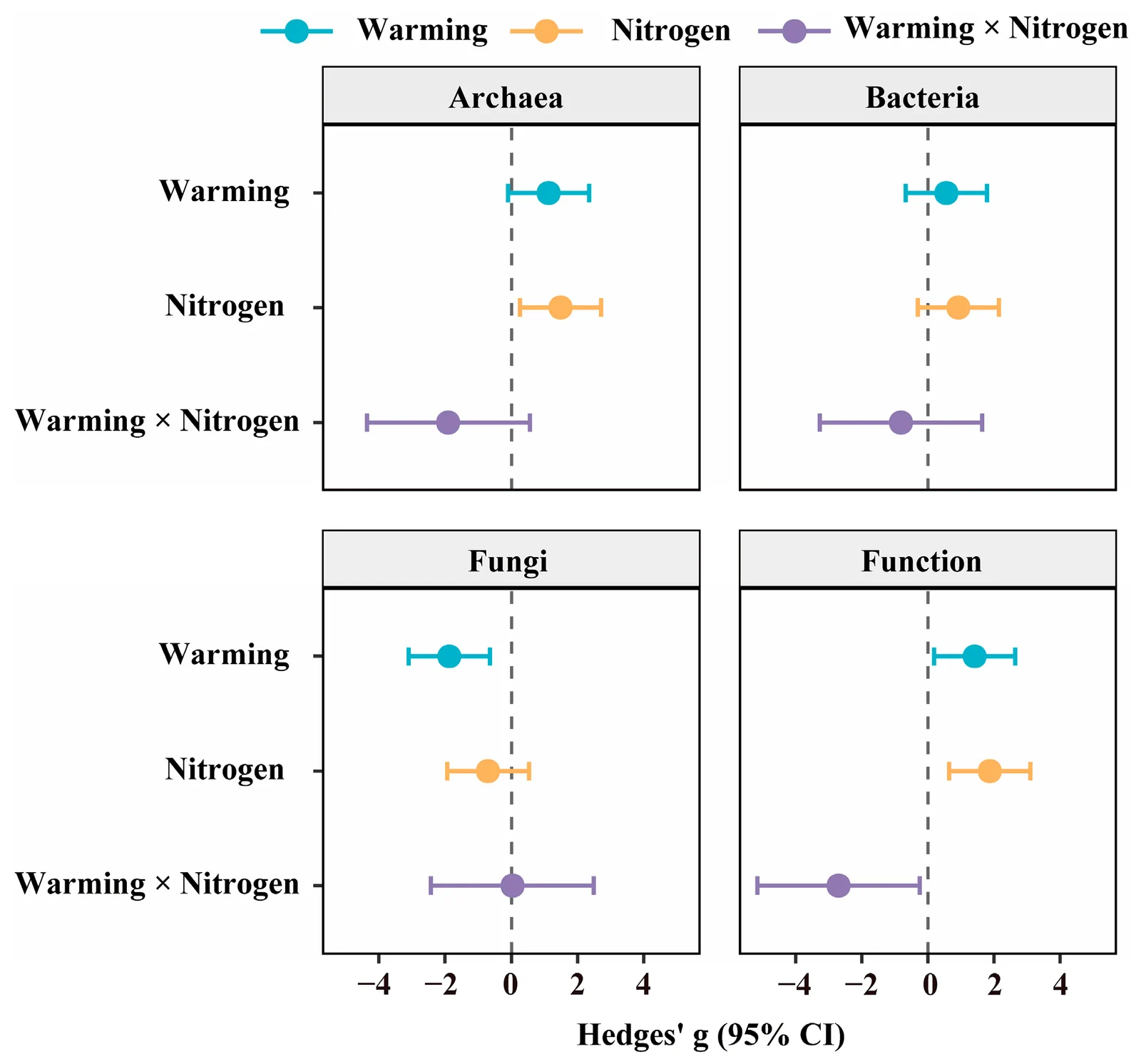
Standardized effect sizes (Hedges’ g) of the main and interactive effects of warming and nitrogen addition on the richness of soil archaeal, bacterial, and fungal communities and functional genes. Points represent mean effect sizes, and error bars indicate 95% confidence interval (CI). Effects were considered significant when the 95% CI did not overlap zero. Positive values indicate increases relative to the control, whereas negative values indicate decreases.

**Figure 5:**
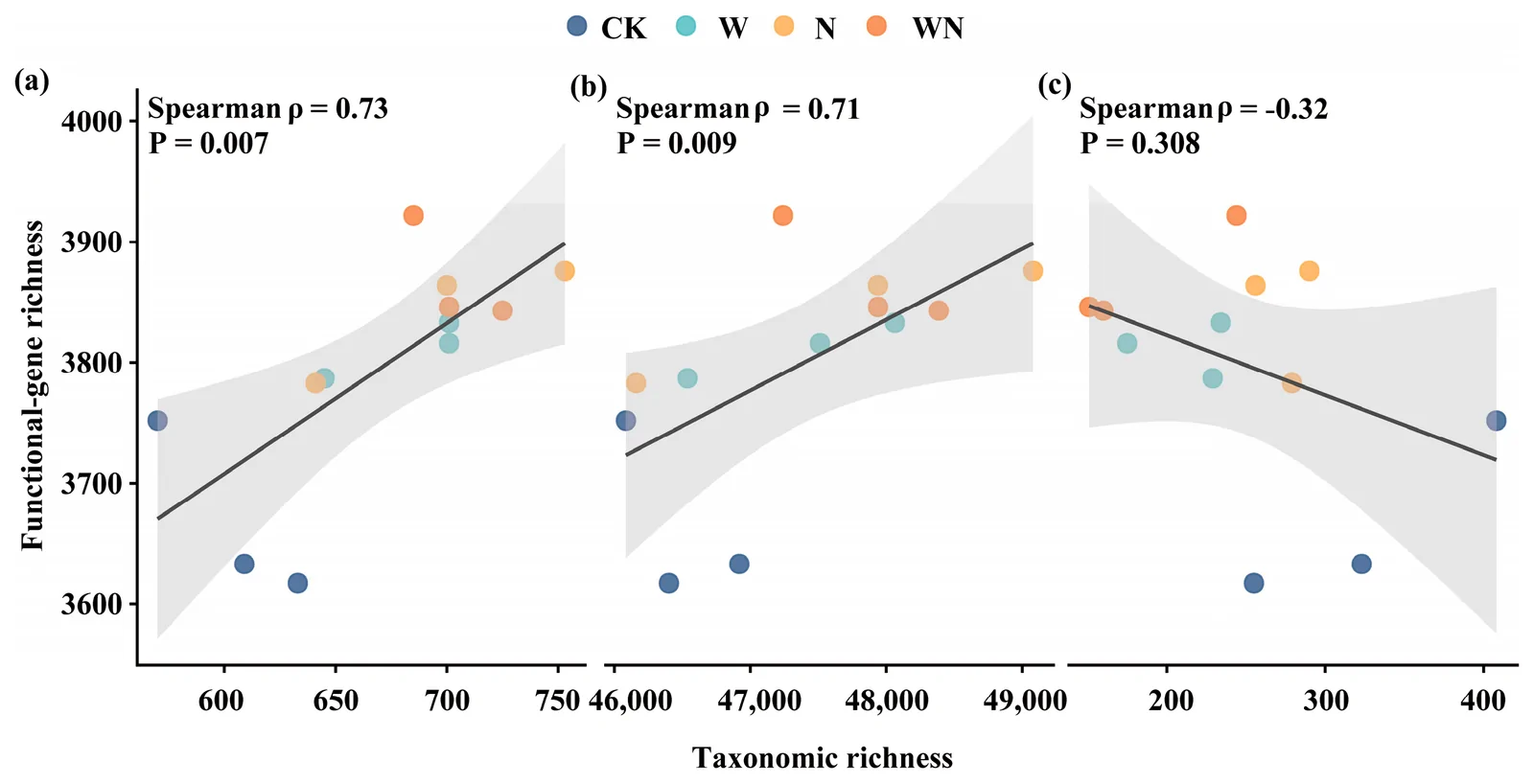
Relationships between functional gene richness and archaeal richness (**a**), bacterial richness (**b**), and fungal richness (**c**) across warming and N-addition treatments. Black lines and grey bands represent ordinary least-squares regression fits and their 95% confidence intervals, which are included only to visualize the overall trends; statistical inference was based on Spearman’s rank correlations. *CK*, control treatment; *W*, warming treatment; *N*, nitrogen addition treatment; *WN*, warming nitrogen addition treatment.

**Figure 6:**
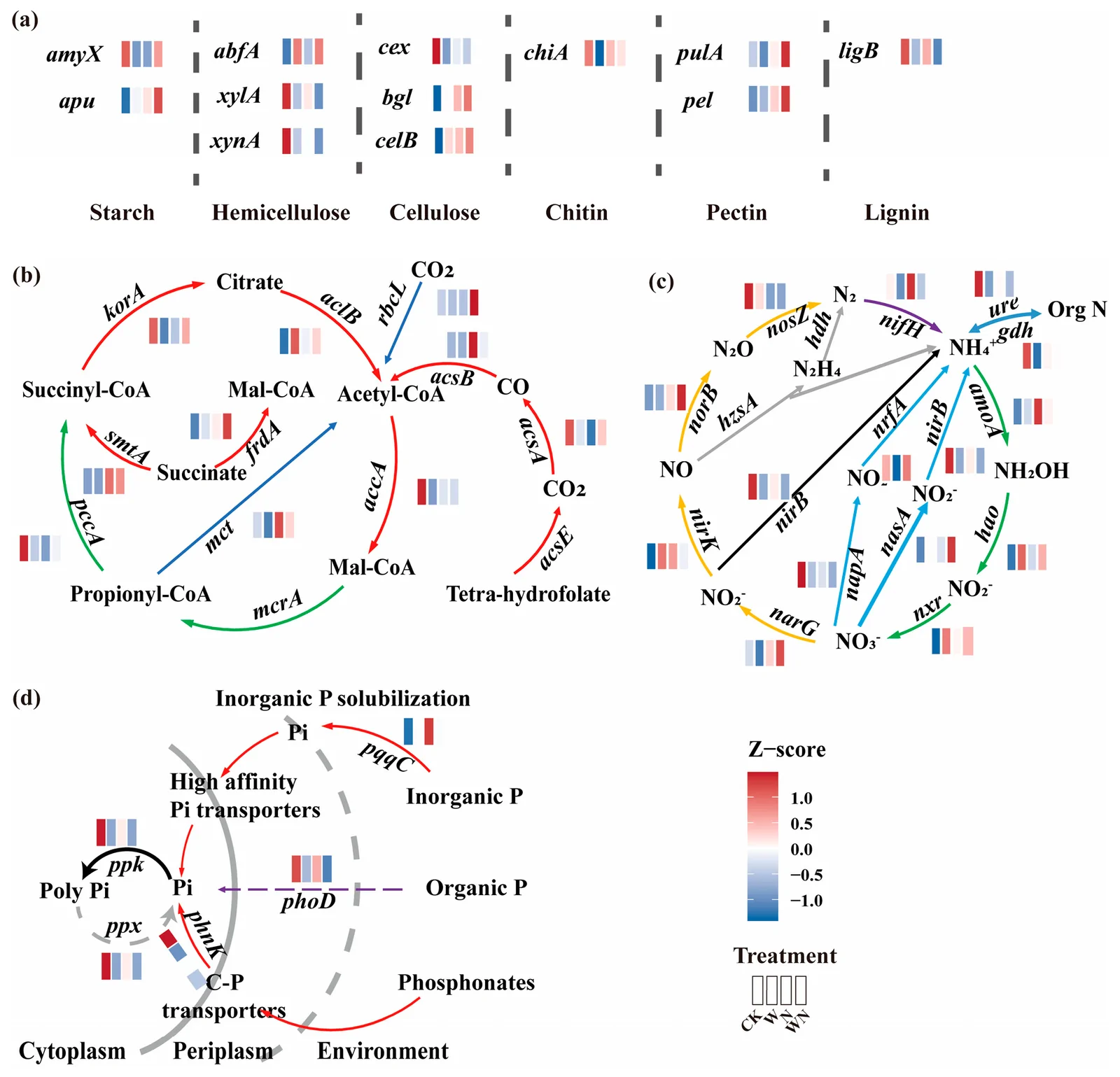
Comparison of microbial metabolism pathways related to soil carbon, nitrogen, and phosphorus cycles under warming and nitrogen addition treatments. Carbon degradation (**a**); carbon fixation (**b**); nitrogen cycle (**c**); phosphorus cycle (**d**). Heatmaps represent the normalized abundance of microbial metabolism pathways under each treatment. *CK*, control treatment; *W*, warming treatment; *N*, nitrogen addition treatment; *WN*, warming nitrogen addition treatment.

**Figure 7:**
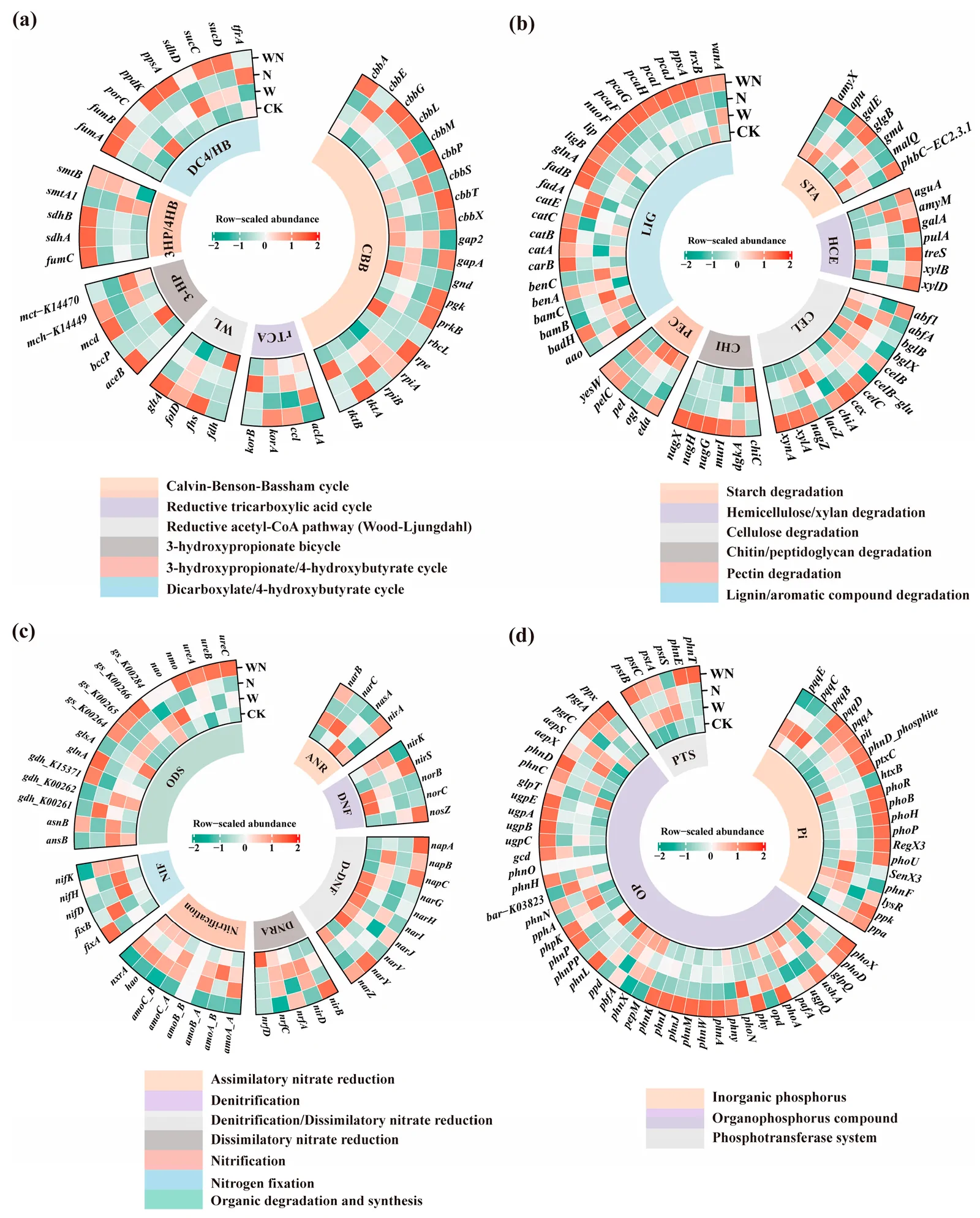
Abundance of microbial functional genes related to soil carbon, nitrogen and phosphorus cycles under warming and N addition treatments. Carbon degradation (**a**); carbon fixation (**b**); nitrogen cycle (**c**); phosphorus cycle (**d**). Heatmaps represent the normalized abundance of functional genes under each treatment. *CK*, control treatment; *W*, warming treatment; *N*, nitrogen addition treatment; *WN*, warming nitrogen addition treatment.

**Figure 8:**
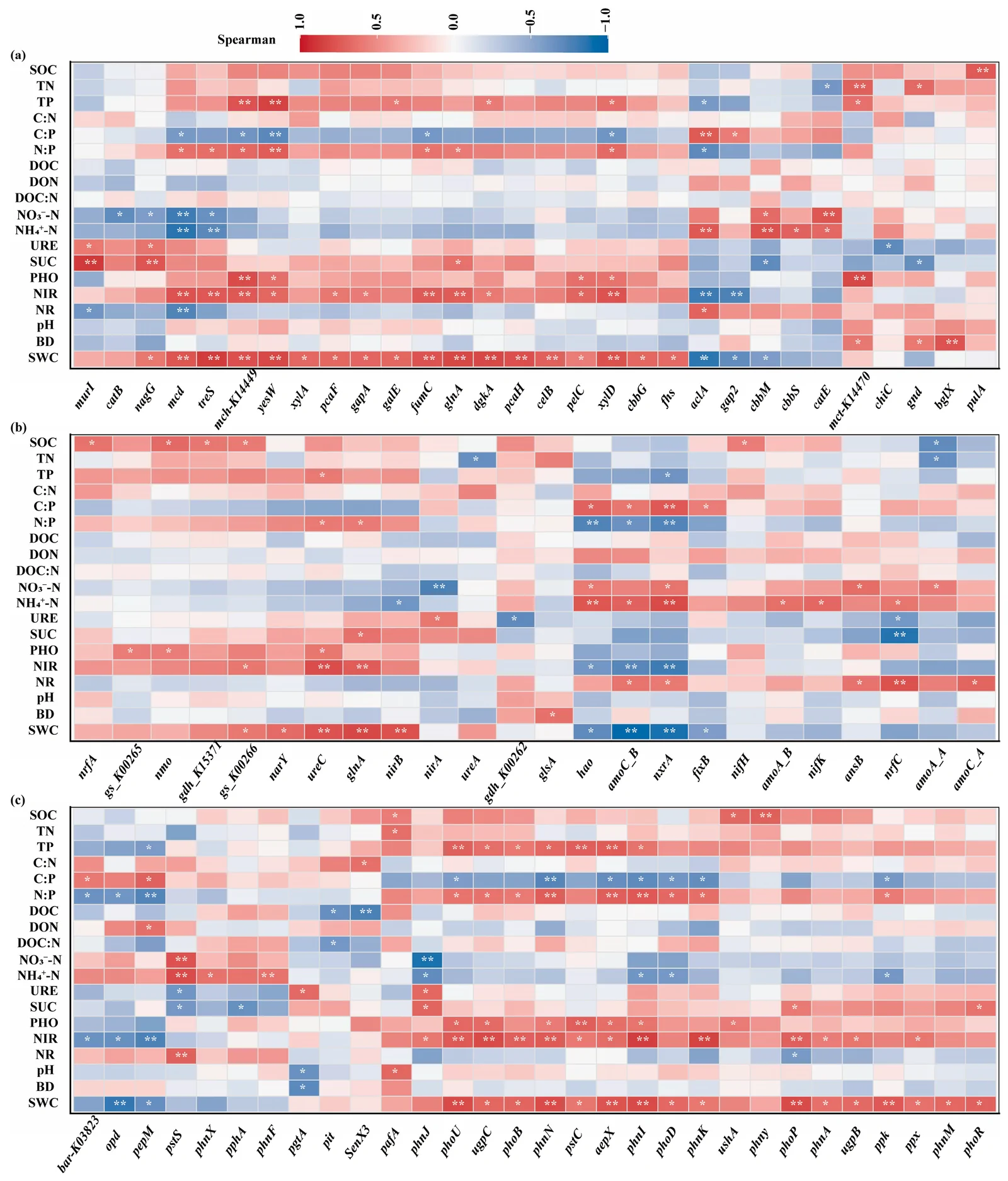
Heatmap of soil physical and chemical factors and the abundance of functional genes for carbon (**a**), nitrogen (**b**) and phosphorus (**c**) cycling. Abundance genes significantly associated with at least one environmental variable are shown. BD, bulk density; SWC, soil water content; SOC, soil organic carbon; TN, total nitrogen; TP, total phosphorus; C:N, carbon to nitrogen ratio; C:P, carbon to phosphorus ratio; N:P, nitrogen to phosphorus ratio; DOC, dissolved organic carbon; DON, dissolved organic nitrogen; DOC:N, dissolved organic carbon to nitrogen ratio; NH_4_^+^-N, ammonium nitrogen; NO_3_^−^-N, nitrate nitrogen; URE, urease activity; SUC, sucrase activity; PHO, phosphatase activity; NR, nitrate reductase activity; NIR, nitrite reductase activity. *, and ** indicate *p* < 0.05, and *p* < 0.01, respectively.

**Figure 9:**
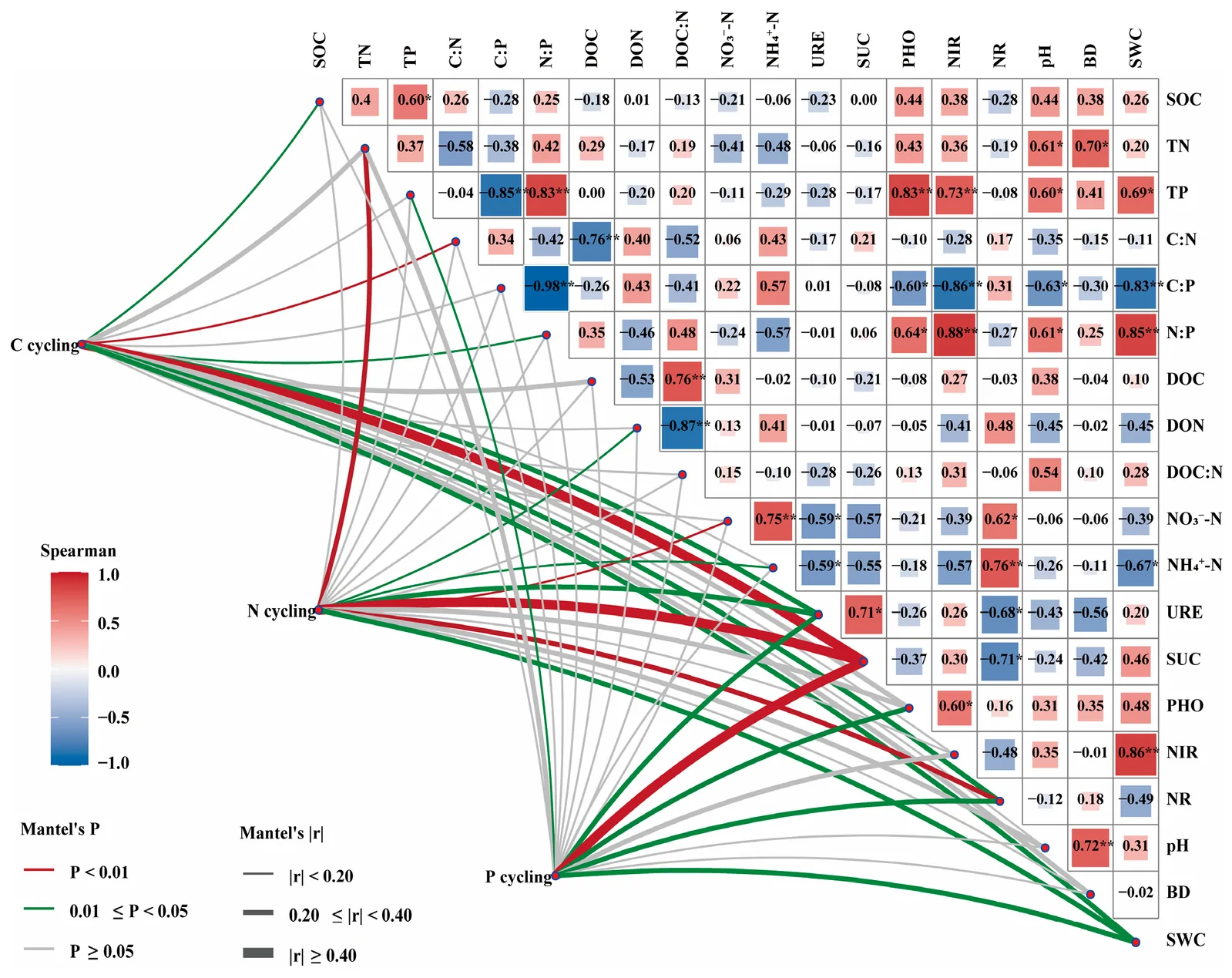
Mantel correlations between C, N and P cycle and biotic and abiotic factors. Red and blue indicate positive and negative correlations, respectively. The size of each square is proportional to the absolute value of the correlation coefficient. *, and **, indicate *p* < 0.05, and *p* < 0.01, respectively. Relationships between carbon, nitrogen and phosphorus cycle and environmental factors were assessed using Mantel tests. Line width corresponds to the Mantel statistic, and line color indicates significance levels based on 9999 permutations.

## Data Availability

The raw shotgun metagenomic sequencing reads generated in this study have been deposited in the Genome Sequence Archive (GSA) under accession number CRA049558. The processed data supporting the findings of this study are included in the article and Supplementary Materials.

## Supplementary Materials

The following supporting information can be downloaded at https://www.mdpi.com/article/10.3390/microorganisms14102151/s1. Refs. [37,45,73,74,75,76] can be found in Supplementary Materials.
