# Supplementary material for: Warming and Nitrogen Addition Induce Asynchronous Taxonomic and Functional Responses and Reorganize Microbial C, N, P Cycling Potential in an Alpine Wetland on the Qinghai–Tibet Plateau
Source: Microorganisms. 2026 Sep 23;14(10):2151. doi: 10.3390/microorganisms14102151 (PMC13707443; doi:10.3390/microorganisms14102151)
Supplement: Supplementary file 1 [file microorganisms-14-02151-s001.zip › microorganisms-4538592-supplementary.pdf]

Supplementary text

Text S1: Soil physicochemical examination

Soil pH was measured in a soil water slurry (1:2.5, soil to water ratio) using PHS-3C meter (Shanghai Leici Instrument, Shanghai, China) [1]. Soil bulk density (BD) was measured using a stainless-steel cutting ring with a volume of 100 cm^3. Soil samples were dried at 105°C for 48 hours to determine soil water content (SWC). Soil temperature (TEM) was measured in situ at 0-10 cm depth of using 5 TM sensor. Soil organic carbon (SOC) was determined using the potassium dichromate oxidation method, total nitrogen (TN) using the Kjeldahl digestion method, and total phosphorus (TP) using the molybdenum–antimony colorimetric method following $HClO_4$-H_2SO_4 digestion methods [2]. Dissolved organic carbon (DOC) and nitrogen (DON) concentration were determined from the extract using a TOC-V analyzer. Soil ammonium nitrogen (NH_4^+ -N) and nitrate nitrogen (NO_3^- -N) content was determined by leaching-distillation of KCl solution method [3]. Soil urease (URE), sucrase (SUC), phosphatase (PHO), nitrate reductase (NR), and nitrite reductase (NIR) activities were determined using chromatography [4]. The determination and calculation of soil enzyme activity is described in detail in Chang et al [5].

Text S2: Calculation method

The effects of the indicators on the control treatment were quantified and compared in terms of response ratios under treatment, using the following equations:

$$lnRR = \ln(\bar{X}_{t}/\bar{X}_{CK})$$

where $\bar{x}_t$ and $\bar{x}_c$ are the mean values of variables in different treatments and control treatment, respectively. Positive response ratio values indicate an increase in metrics for that treatment group, while negative values indicate the opposite trend.

Furthermore, Category-functional response differences:

$$\Delta lnRR = lnRR_{(Function)} - lnRR_{(Taxon)}$$

An index of 0 indicates the same magnitude of categorical and functional responses, a positive value indicates a more positive response for functional Richness than categorical Richness, and a negative value indicates a stronger response for categorical Richness.

Main effect and interaction effect

To assess the extent to which individual effects, main effects, and their interaction effects, we used the Hedges'g test. We calculated the main effect of climate warming (L_W), the main effect of nitrogen addition (L_N), and their interaction effects (L_I), respectively. $\bar{X}_{CK}$, $\bar{X}_W$, $\bar{X}_N$ *and* $\bar{X}_{WN}$ are used to represent the mean values of CK, W, N and WN, respectively.

Main effect of warming:

$$L_W = \frac{\bar{X}_W + \bar{X}_{WN}}{2} - \frac{\bar{X}_{CK} + \bar{X}_N}{2}$$

Main effect of nitrogen addition:

$$L_N = \frac{\bar{X}_N + \bar{X}_{WN}}{2} - \frac{\bar{X}_{CK} + \bar{X}_W}{2}$$

Interaction effects:

$$L_I = (\bar{X}_{WN} - \bar{X}_W) - (\bar{X}_N - \bar{X}_{CK})$$

Sum expected value:

$$\bar{X}_{WN,add} = \bar{X}_W + \bar{X}_N - \bar{X}_{CK}$$

$$L_{WN} = \bar{X}_{WN} - \bar{X}_{WN,add}$$

The interaction was calculated as the difference in differences, the deviation between the observed response of WN and warming and nitrogen addition and expectation.

We classified interaction effects as significant (antagonistic or synergistic) and nonsignificant (additive) using Hedges' g calculated interaction effects and 95% confidence interval effect sizes. If the 95% CI overlaps to zero, the interaction effect was considered additive. Interaction > 0 indicates antagonism (< 0 indicates cooperativity) when individual effects are in opposite directions or both are negative. If both individual effects are positive, an interaction > 0 indicates synergy (< 0 indicates antagonism) [6].

References

1. Bao, S.D. *Soil and agricultural chemistry analysis*; china agriculture science and technique press: Beijing, 2000;
2. Lu, R.K. *Methods of soil agricultural chemical analysis*; chinese agricultural science and technology press: Beijing, 2000;
3. Riley, W.J.; Ortiz-Monasterio, I.; Matson, P.A. Nitrogen leaching and soil nitrate, nitrite, and ammonium levels under irrigated wheat in northern mexico. *Nutr. Cycling Agroecosyst.* **2001**, *61*, 223–236, doi:10.1023/A:1013758116346.
4. Guan, S.Y.; Zhang, D.; Zhang, Z. *Soil enzyme and its research methods*; Agricultural Press: Beijing, 1986;
5. Chang, W.; Ma, W.; Song, L.; Tang, Y.; Long, Y.; Xu, G.; Yuan, J. Responses of soil n-cycle enzyme activities to vegetation degradation in a wet meadow on the qinghai-tibet plateau. *Front. Ecol. Evol.* **2023**, *11*, 1210643, doi:10.3389/fevo.2023.1210643.
6. Hedges, L.V.; Gurevitch, J.; Curtis, P.S. The meta-analysis of response ratios in experimental ecology. *Ecology* **1999**, *80*, 1150–1156, doi:10.1890/0012-9658(1999)080[1150:TMAORR]2.0.CO;2.

Table

Table S1: Basic information of metagenomic sequencing.

Sample	Clean reads (M)	Percent in raw reads (%)[a]	Contigs	ORFs[b]	N50[c]	N90[d]
CK1	79.10	97.64	263,222	380,956	712	532
CK2	74.40	97.79	282,137	419,297	738	535
CK3	74.94	97.49	291,067	429,762	756	537
W1	73.04	97.83	356,648	537,553	783	540
W2	77.06	97.84	321,202	472,878	744	536
W3	77.91	97.61	316,043	465,900	751	537
N1	78.10	97.92	318,823	469,289	747	536
N2	70.88	97.43	267,340	390,140	729	534
N3	71.03	97.56	333,589	497,023	766	539
WN1	76.10	97.75	340,354	508,778	762	538
WN2	74.91	97.88	346,059	515,341	774	539
WN3	69.27	97.78	322,844	481,545	767	539

Note: CK, control treatment; W, warming treatment; N, nitrogen addition treatment; WN, warming nitrogen addition treatment

[a] The percentage of clean read in its corresponding raw read.

[b] ORFs, Open reading frames.

[c] N50 represents the length of the contig overlapping the midpoint of the length-order concatenation of contigs.

[d] N90 represents the length of the contig overlapping the ninety percent of the length-order concatenation of contigs.

Table S2: Information on microbial functional genes involved in the C cycling processes identified in this study.

Type	KEGG number	Encoded protein (EC)	Gene name
		**3-hydroxypropionate**	
	K01638	2.3.3.9	*aceB*
	K02160	6.4.1.2	*bccP*
	K14448	1.3.8.12	*mcd*
	K14449	4.2.1.148	*mch*
	K14470	5.4.1.3	*mct*
		**3-hydroxypropionate/4-hydroxybutyrate**	
	K01679	4.2.1.2	*fumC*
	K00239	1.3.5.1	*sdhA*
	K00240	1.3.5.1	*sdhB*
	K14471	2.8.3.22	*smtA1*
	K14472	2.8.3.22	*smtB*
		**Calvin Benson Bassham cycle**	
	K01624	4.1.2.13	*cbbA*
	K01783	5.1.3.1	*cbbE*
	K00134	1.2.1.12	*cbbG*
	K01601	4.1.1.39	*cbbL*
	K01601	4.1.1.39	*cbbM*
	K00855	2.7.1.19	*cbbP*
Carbon fixation	K01602	4.1.1.39	*cbbS*
	K00615	2.2.1.1	*cbbT*
	K06413	—	*cbbX*
	K00150	1.2.1.59	*gap2*
	K00134	1.2.1.12	*gapA*
	K00033	1.1.1.44/1.1.1.343	*gnd*
	K00927	2.7.2.3	*pgk*
	K00855	2.7.1.19	*prkB*
	K01601	4.1.1.39	*rbcL*
	K01783	5.1.3.1	*rpe*
	K01807	5.3.1.6	*rpiA*
	K01808	5.3.1.6	*rpiB*
	K00615	2.2.1.1	*tktA*
	K00615	2.2.1.1	*tktB*
		**Dicarboxylate/4-hydroxybutyrate**	
	K01677	4.2.1.2	*fumA*
	K01678	4.2.1.2	*fumB*
	K00172	1.2.7.1	*porC*
	K01006	2.7.9.1	*ppdK*
	K01007	2.7.9.2	*ppsA*
	K00242	1.3.5.1	*sdhD*

	K01903	6.2.1.5	*sucC*
	K01902	6.2.1.5	*sucD*
	K18209	1.3.4.1	*tfrA*
		**Reductive tricarboxylic acid cycle**	
	K15230	2.3.3.8	*aclA*
	K15234	4.1.3.34	*ccl*
	K00174	1.2.7.3	*korA*
	K00175	1.2.7.11	*korB*
		**Wood Ljungdahl pathway**	
	K05299	1.17.1.9	*fdh*
	K01938	6.3.4.3	*fhs*
	K01491	1.5.1.5/3.5.4.9	*folD*
	K01647	2.3.3.1	*gltA*
Carbon degradation		**Cellulose**	
	K05350	3.2.1.21	*bglB*
	K05349	3.2.1.21	*bglX*
	K19357	3.2.1.4	*celB*
	K01835	5.4.2.2	*celB-glu*
	K02759	2.7.1.196/2.7.1.205	*celC*
	K00170	1.2.7.1	*cex*
	K01190	3.2.1.23	*lacZ*
	K01207	3.2.1.52	*nagZ*
	K01805	5.3.1.5	*xylA*
	K01181	3.2.1.8	*xynA*
		**Chitin**	
	K13381	3.2.1.14/3.2.1.17	*chiA*
	K01183	3.2.1.14	*chiC*
	K00887	2.7.1.66	*dgkA*
	K01776	5.1.1.3	*murI*
	K18242	1.14.13.172	*nagG*
	K18243	1.14.13.172	*nagH*
	K22270	1.14.13.24	*nagX*
		**Hemicellulose**	
	K20844	3.2.1.55	*abf1*
	K01209	3.2.1.55	*abfA*
	K05992	3.2.1.133	*amyM*
	K04099	1.13.11.57	*galA*
	K01200	3.2.1.41	*pulA*
	K05343	5.4.99.16	*treS*
	K00854	2.7.1.17	*xylB*
	K22186	4.2.1.82	*xylD*

	**Lignin**	
K00273	1.1.3.7	*aao*
K07535	1.1.1.-	*badH*
K19515	1.3.-.-	*bamB*
K19516	1.3.-.-	*bamC*
K05549	1.14.12.10	*benA*
K05784	1.14.12.10	*benC*
K26912	4.1.99.28	*carB*
K03381	1.13.11.1	*catA*
K01856	5.5.1.1	*catB*
K03464	5.3.3.4	*catC*
K07104	1.13.11.2	*catE*
K00632	2.3.1.16	*fadA*
K00074	1.1.1.157	*fadB*
K20712	5.4.4.3	*glnA*
K04101	1.13.11.8	*ligB*
K23515	1.11.1.14	*lip*
K00335	7.1.1.2	*nuoF*
K07823	2.3.1.174	*pcaF*
K00448	1.13.11.3	*pcaG*
K00449	1.13.11.3	*pcaH*
K01031	2.8.3.6	*pcaI*
K01032	2.8.3.6	*pcaJ*
K01007	2.7.9.2	*ppsA*
K00384	1.8.1.9	*trxB*
K03862	1.14.13.82	*vanA*
	**Pectin**	
K01625	4.1.2.14/4.1.3.42	*eda*
K01730	4.2.2.6	*ogl*
K01728	4.2.2.2	*pel*
K19551	4.2.2.2/4.2.2.10	*pelC*
K18197	4.2.2.23	*yesW*
	**Starch**	
K01200	3.2.1.41	*amyX*
K01200	3.2.1.41	*apu*
K01784	5.1.3.2	*galE*
K00700	2.4.1.18	*glgB*
K01711	4.2.1.47	*gmd*
K00705	2.4.1.25	*malQ*
K03821	2.3.1	*phbC*

Table S3: Information on microbial functional genes involved in the N cycling processes identified in this study.

KEGG number	Encoded protein (EC)	Gene name
**Assimilatory nitrate reduction**		
K00367	1.7.7.2	*narB*
K15879	—	*narC*
K00372	1.7.99.-	*nasA*
K00366	1.7.7.1	*nirA*
**Denitrification**		
K00368	1.7.2.1	*nirK*
K15864	1.7.99.1	*nirS*
K04561	1.7.2.5	*norB*
K02305	1.7.2.5	*norC*
K00376	1.7.2.4	*nosZ*
**Denitrification/Dissimilatory nitrate reduction**		
K02567	1.9.6.1	*napA*
K02568	—	*napB*
K02569	—	*napC*
K00370	1.7.5.1/1.7.99.-	*narG*
K00371	1.7.5.1/1.7.99.-	*narH*
K00374	1.7.5.1/1.7.99.-	*narI*
K00373	—	*narJ*
K00374	1.7.5.1 1.7.99.-	*narV*
K00371	1.7.5.1 1.7.99.-	*narY*
K00370	1.7.5.1 1.7.99.-	*narZ*
**Dissimilatory nitrate reduction**		
K00362	1.7.1.15	*nirB*
K00363	1.7.1.15	*nirD*
K03385	1.7.2.2	*nrfA*
K04014	—	*nrfC*
K04015	—	*nrfD*
**Nitrification**		
K28504	1.14.99.39	*amoA_A*
K28504	1.14.99.39	*amoA_B*
K10945	1.14.18.3	*amoB_A*
K10945	1.14.18.3	*amoB_B*
K10946	1.14.18.3	*amoC_A*
K10946	1.14.18.3	*amoC_B*
K10535	1.7.2.6	*hao*
K00370	1.7.5.1 1.7.99.-	*nxrA*
**Nitrogen fixation**		
K03521	—	*fixA*
K03522	—	*fixB*
K02586	1.18.6.1	*nifD*

K02588	1.18.6.1	*nifH*
K02591	1.18.6.1	*nifK*
	**Organic degradation and synthesis**	
K01424	3.5.1.1	*ansB*
K01953	6.3.5.4	*asnB*
K00261	1.4.1.3	*gdh_K00261*
K00262	1.4.1.4	*gdh_K00262*
K15371	1.4.1.2	*gdh_K15371*
K01915	6.3.1.2	*glnA*
K01425	3.5.1.2	*glsA*
K00264	1.4.1.14	*gs_K00264*
K00265	1.4.1.13	*gs_K00265*
K00266	1.4.1.13	*gs_K00266*
K00284	1.4.7.1	*gs_K00284*
K19823	1.7.3.1	*nao*
K00459	1.13.12.16	*nmo*
K01430	3.5.1.5	*ureA*
K01429	3.5.1.5	*ureB*
K01428	3.5.1.5	*ureC*

Table S4: Information on microbial functional genes involved in the P cycling processes identified in this study.

KEGG number	Encoded protein (EC)	Gene name
	**Inorganic phosphorus**	
K06139	1.21.98.4	*pqqE*
K06137	1.3.3.11	*pqqC*
K06136	—	*pqqB*
K06138	—	*pqqD*
K06135	—	*pqqA*
K16322	—	*pit*
—	—	*ptxC*
—	—	*htxB*
K07636	2.7.13.3	*phoR*
K07657	—	*phoB*
K06217	—	*phoH*
K07658	—	*phoP*
K07776	—	*RegX3*
K02039	—	*phoU*
K07768	2.7.13.3	*SenX3*
K02043	—	*phnF*
K08476	—	*pgtA*
K01507	3.6.1.1	*ppa*
K01524	3.6.1.11/3.6.1.40	*ppx*
	**Organophosphorus compound**	
K07093	**3.1.3.1**	*phoX*
K01113	3.1.3.1	*phoD*
K01126	3.1.4.46	*glpQ*
K11751	3.1.3.5/3.6.1.45	*ushA*
K01126	3.1.4.46	*ugpQ*
K07194	6.3.1.19	*pafA*
K01077	3.1.3.1	*phoA*
K07048	3.1.8.1	*opd*
K01083	3.1.3.8	*phy*
K09474	3.1.3.2	*phoN*
K21195	1.14.11.46	*phnY*
K19670	3.11.1.2	*phnA*
K03430	2.6.1.37	*phnW*
K06162	3.6.1.63	*phnM*
K06163	4.7.1.1	*phnJ*
K06164	2.7.8.37	*phnI*
K05781	—	*phnK*
K01841	5.4.2.9	*pepM*
K05306	3.11.1.1	*phnX*

K26913	4.3.1.33	*pbfA*
K09459	4.1.1.82	*ppd*
K05780	2.7.8.37	*phnL*
K20859	3.1.4.57	*phnPP*
K06167	3.1.4.55	*phnP*
K12914	2.1.1.326	*phpK*
K19669	3.11.1.3	*pphA*
K05774	2.7.4.23	*phnN*
K03823	2.3.1.183	*bar*
K06165	2.7.8.37	*phnH*
K09994	2.3.1.280/2.3.1.338	*phnO*
K00117	1.1.5.2	*gcd*
K05816	7.6.2.10	*ugpC*
K05813	—	*ugpB*
K05814	—	*ugpA*
K05815	—	*ugpE*
K02445	—	*glpT*
K02041	7.3.2.2	*phnC*
K02044	—	*phnD*
K01841	—	*aepX*
—	—	*aepS*
K08478	—	*pgtC*
K00937	2.7.4.1	*ppk*
	**Phosphotransferase system**	
K02036	7.3.2.1	*pstB*
K02037	—	*pstC*
K02038	—	*pstA*
K02040	—	*pstS*
K02042	—	*phnE*
K11084	—	*phnT*

Table S5: Basic chemical properties of the soils and plants from the four treatments (Mean ±SE).

Variable	CK	W	N	WN
AGB (g m^2)	118.73±14.86b	161.03±9.39ab	158.95±22.05ab	182.30±20.85a
BGB (g m^2)	1554.90±8.30bc	2258.90±44.36a	1668.60±27.60b	1533.90±22.90c
TB (g m^2)	1673.63±23.06c	2419.89±50.37a	1827.54±49.02b	1716.17±43.36bc
pH	7.63±0.02a	7.34±0.06b	7.66±0.01a	7.67±0.02a
BD ($g \cdot cm^{-3}$)	0.93±0.07b	0.92±0.07b	1.19±0.01a	1.19±0.06a
SWC (m^{-3} m^{-3})	0.46±0.01a	0.39±0.01b	0.42±0.00ab	0.39±0.01b
TEM (℃)	11.13±0.22b	12.10±0.16a	11.65±0.12b	12.62±0.26a
SOC (g kg^{-1})	37.23±3.53ab	30.97±0.46b	40.55±1.88a	41.14±0.40a
TN (g kg^{-1})	1.21±0.04ab	0.88±0.13b	1.24±0.03ab	1.41±0.09a
TP (g kg^{-1})	0.71±0.00a	0.20±0.02c	0.74±0.00a	0.36±0.02b
C:N	30.87±3.39a	36.67±5.38a	32.87±2.18c	29.44±2.16b
C:P	42.36±0.87c	160.32±14.19a	54.97±2.74c	115.21±5.66b
N:P	1.71±0.06b	4.45±0.28a	1.68±0.04b	3.98±0.47a
DOC (mg kg^{-1})	34.06±6.84a	32.98±4.35a	35.28±0.35a	34.43±3.59a
DON (mg kg^{-1})	3.03±0.30b	3.74±0.29a	3.26±0.33b	3.34±0.37b
DOC: N	11.87±3.20a	9.03±1.65a	12.37±3.45a	11.17±3.95a
NH_4^+-N ($mg \cdot kg^{-1}$)	0.72±0.07b	1.17±0.19a	1.04±0.11a	0.96±0.10a
NO_3^--N ($mg \cdot kg^{-1}$)	0.61±0.01c	1.19±0.14a	1.08±0.12a	0.80±0.21b
URE ($mg \cdot g^{-1} \cdot d^{-1}$)	1.49±0.03a	0.97±0.00b	0.60±0.09c	0.99±0.10b
SUR ($mg \cdot g^{-1} \cdot d^{-1}$)	54.86±1.32a	29.57±4.63b	23.36±0.35b	30.10±2.02b
PHO ($mg \cdot g^{-1} \cdot d^{-1}$)	0.12±0.01b	0.08±0.02b	0.19±0.01a	0.10±0.01b
NR ($mg \cdot g^{-1} \cdot d^{-1}$)	1.11±0.18b	3.65±0.57a	3.92±0.05a	2.49±0.23ab
NIR ($\mu g \cdot g^{-1} \cdot d^{-1}$)	108.33±1.60a	31.31±0.17d	63.49±0.46b	43.50±1.06c

Note: AGB, aboveground biomass; BGB, belowground biomass; TB, total biomass; BD, bulk density; SWC, soil water content; TEM, temperature; SOC, soil organic carbon; TN, total nitrogen; TP, total phosphorus; C:N, carbon to nitrogen ratio; C:P, carbon to phosphorus ratio; N:P, nitrogen to phosphorus ratio; DOC, dissolved organic carbon; DON, dissolved organic nitrogen; DOC:N, dissolved organic carbon to nitrogen ratio; NH4-N, ammonium nitrogen; NO- 3-N, nitrate nitrogen; URE, urease activity; SUR, sucrase activity; PHO, phosphatase activity; NR, nitrate reductase activity; NIR, nitrite reductase activity. CK, control treatment; W, warming treatment; N, nitrogen addition treatment; WN, warming nitrogen addition treatment. Different lowercase letters indicate significant difference between treatments ($P < 0.05$).

Table S6: Results of a linear mixed model analysis of warming and nitrogen on soil microbial community.

Richness	Effect	F-value	P-value
Archaea	Warming	4.98	0.07
	Nitrogen	8.72	0.03
	Warming*Nitrogen	3.59	0.11
Bacteria	Warming	1.23	0.31
	Nitrogen	3.37	0.12
	Warming*Nitrogen	0.66	0.45
Fungi	Warming	13.83	0.01
	Nitrogen	1.94	0.21
	Warming*Nitrogen	0.06	0.88
Function	Warming	6.93	0.03
	Nitrogen	13.91	0.01
	Warming*Nitrogen	7.58	0.04

Table S7: Results of a linear mixed model analysis of warming and nitrogen on soil cycling.

Richness	Effect	F-value	P-value
Carbon cycle	Warming	4.58	0.07
	Nitrogen	1.03	0.34
	Warming*Nitrogen	1.60	0.24
Nitrogen cycle	Warming	8.23	0.01
	Nitrogen	4.35	0.04
	Warming*Nitrogen	0.39	0.39
Phosphorus cycle	Warming	13.83	0.01
	Nitrogen	1.76	0.22
	Warming*Nitrogen	1.68	0.23

Figure

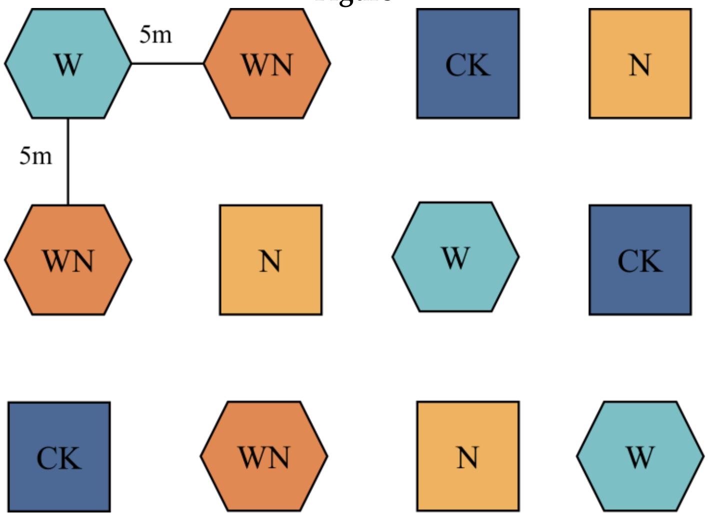

Figure S1: Schematic diagram of experimental treatment design. CK, control treatment; W, warming treatment; N, nitrogen addition treatment; WN, warming nitrogen addition treatment.

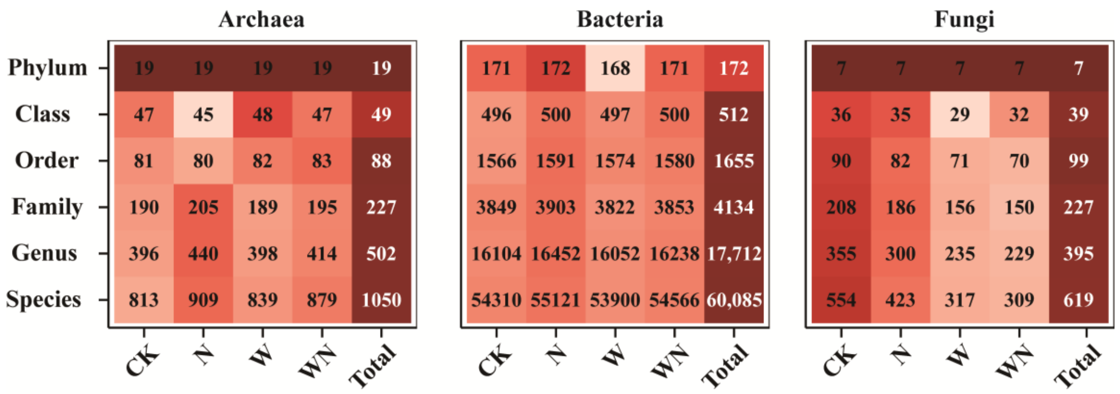

Figure S2: Community composition of phylum, class, order, family, genus and species in their respective treatments and in all treatments at different taxonomic levels. CK, control treatment; W, warming treatment; N, nitrogen addition treatment; WN, warming nitrogen addition treatment.

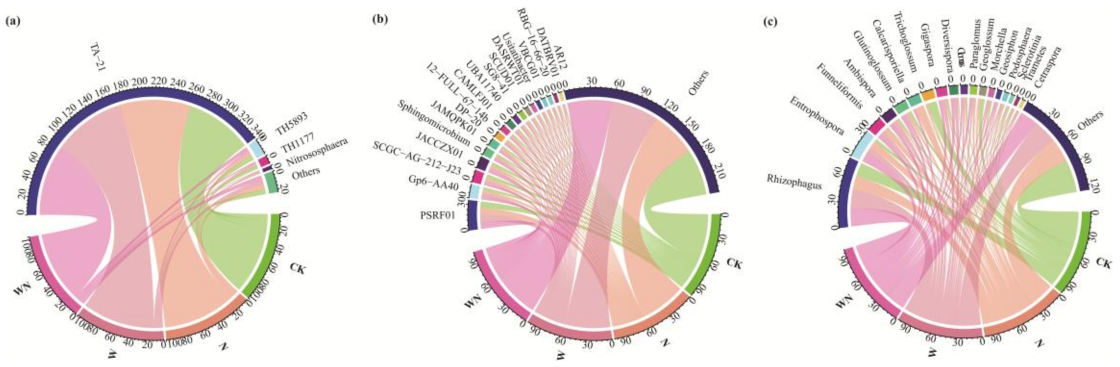

Figure S3: The circos diagram visually demonstrates the similarities and differences between the components. The bacterial genus (a), fungal genus (b), and archaeal genus (c) are treated differently. In the diagram, the outer circles indicate the different microbial genuses, while the inner circles indicate their coexistence relationships. CK, control treatment; W, warming treatment; N, nitrogen addition treatment; WN, warming nitrogen addition treatment.

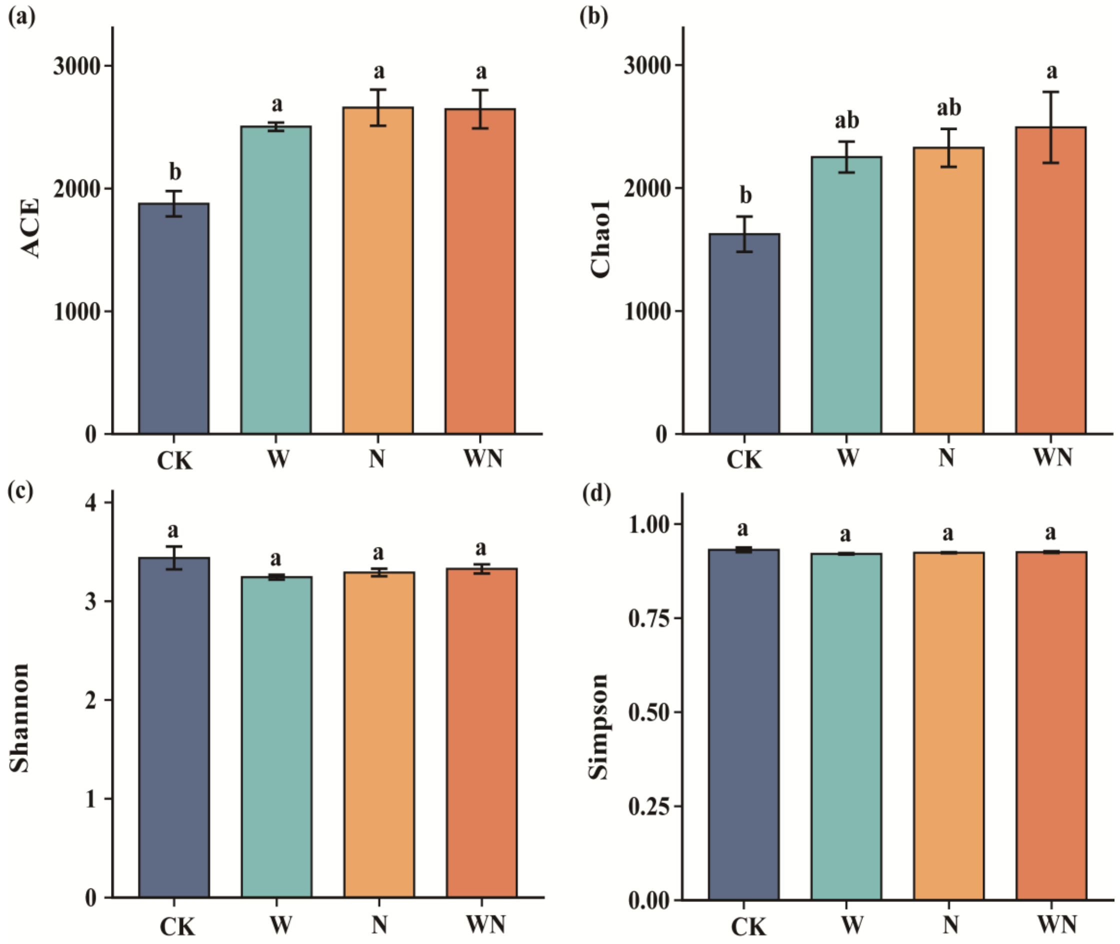

Figure S4: The diversity indices ACE (a), Chao1 (b), Shannon (c), and Simpson (d) of soil archaeal communities under different treatments. CK: control treatment; W: warming treatment; N: nitrogen addition treatment; WN: warming nitrogen addition treatment. Different lowercase letters indicate significant difference between treatments ($P < 0.05$).

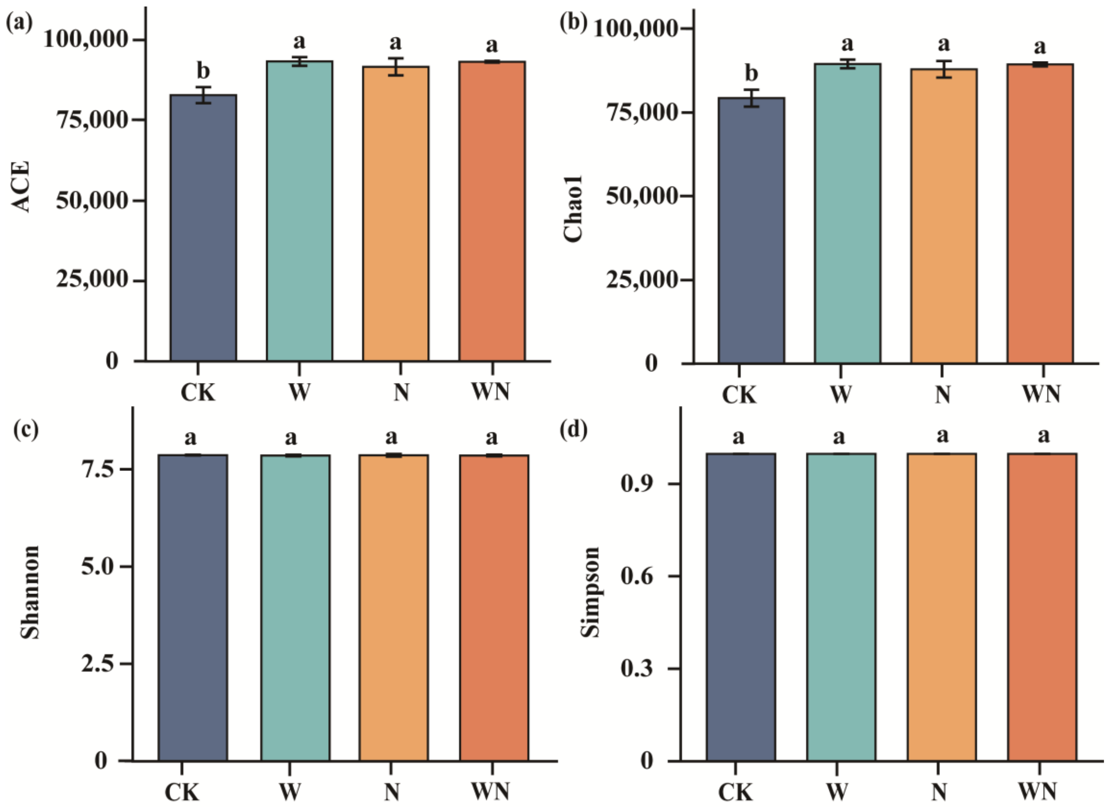

Figure S5: The diversity indices ACE (a), Chao1 (b), Shannon (c), and Simpson (d) of soil bacterial communities under different treatments. CK: control treatment; W: warming treatment; N: nitrogen addition treatment; WN: warming nitrogen addition treatment. Different lowercase letters indicate significant difference between treatments ($P < 0.05$).

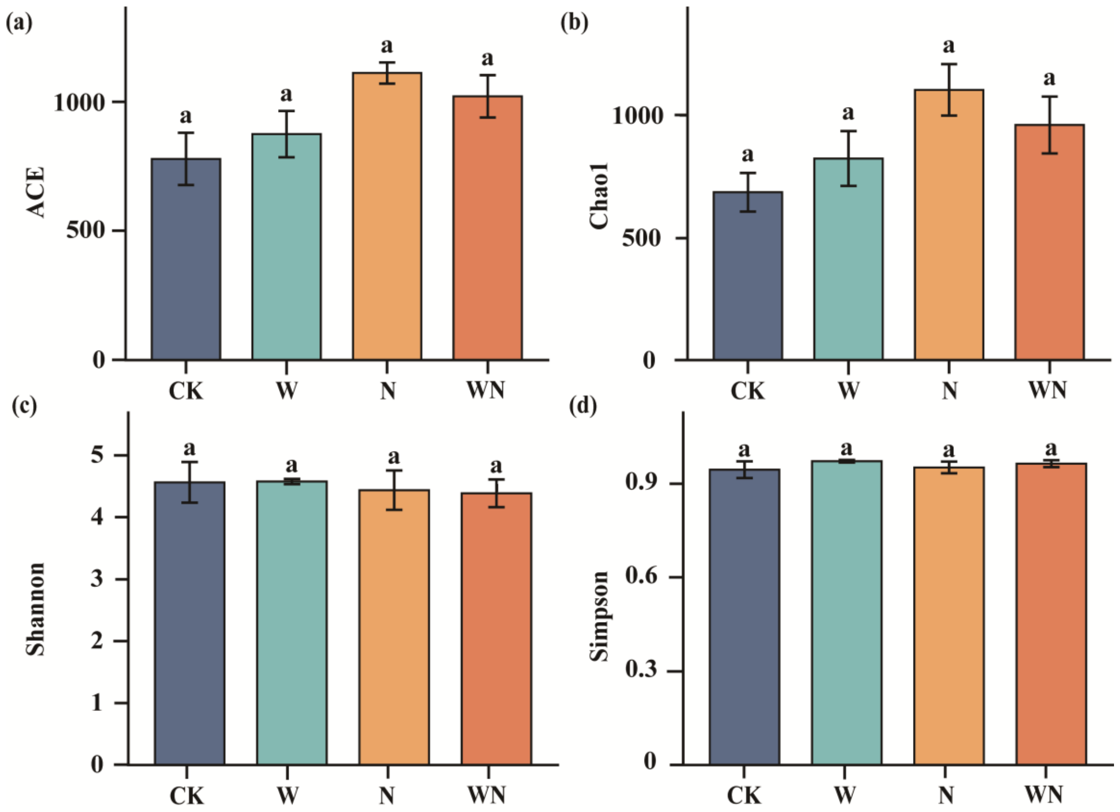

Figure S6: The diversity indices ACE (a), Chao1 (b), Shannon (c), and Simpson (d) of soil fungal communities under different treatments. CK: control treatment; W: warming treatment; N: nitrogen addition treatment; WN: warming nitrogen addition treatment. Different lowercase letters indicate significant difference between treatments ($P < 0.05$).

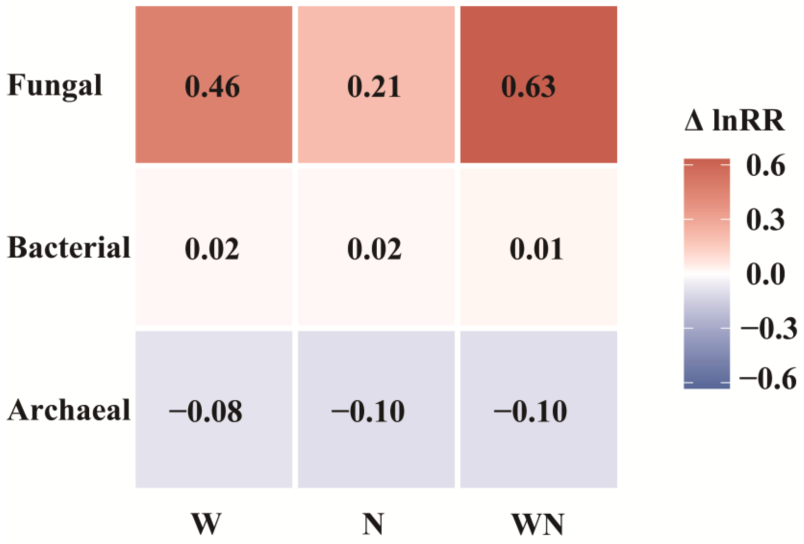

Figure S7: Differences in microbial taxonomic richness versus functional gene richness responses under warming and nitrogen addition. Heatmaps represent lnRR differences in taxonomic richness and functional gene richness of archaea, bacteria, and fungi in response to different treatment magnitudes. Positive values (red) indicate that the response of functional gene richness is more positive or weakly negative than the corresponding taxonomic richness; negative values (blue) indicate that the positive response of taxonomic richness is stronger than functional gene richness; Close to zero indicates that both have similar relative response amplitudes. W: warming treatment; N: nitrogen addition treatment; WN: warming nitrogen addition treatment.

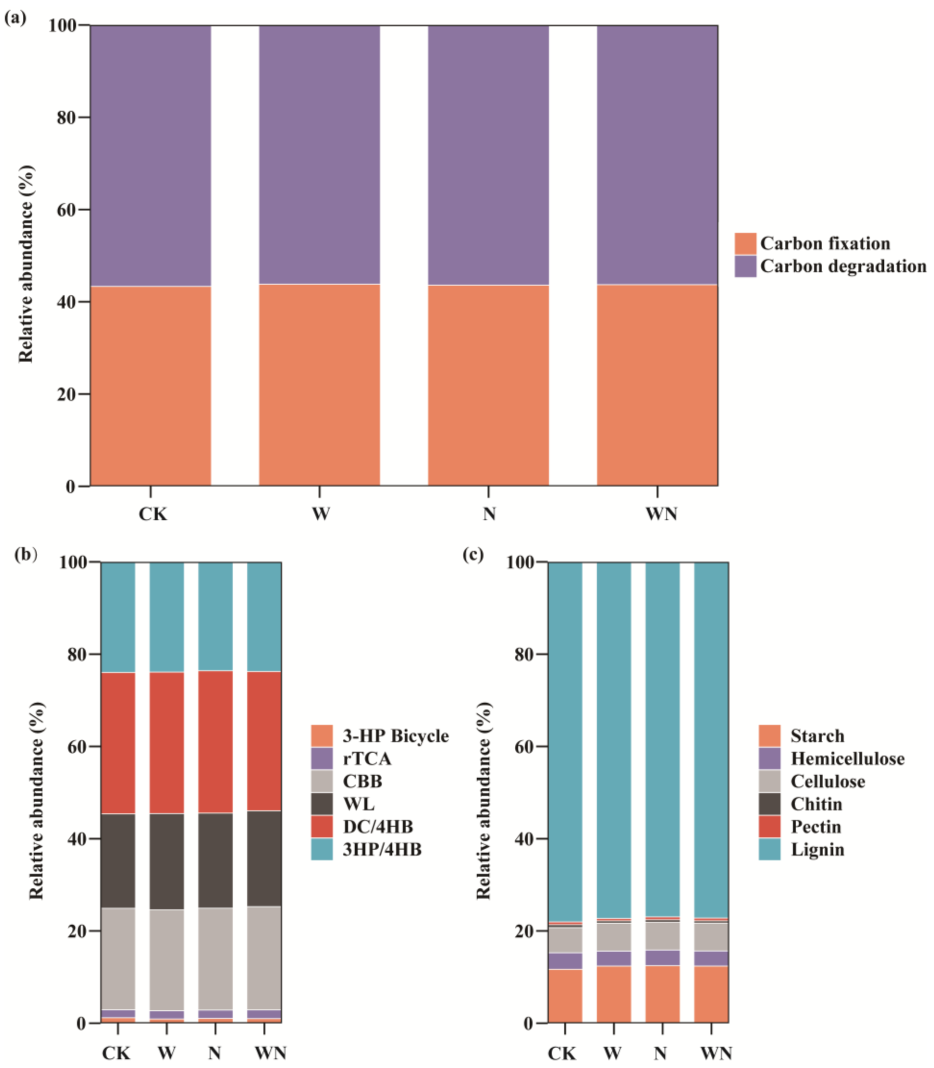

Figure S8: Effects of warming and nitrogen addition on the relative composition of soil microbial carbon cycle functional genes. Relative proportion of carbon fixation versus carbon degradation functional genes in the total abundance of carbon cycle functional genes (a); Relative composition of functional genes in different carbon fixation pathways; (c) Relative composition of functional genes involved in the degradation of different organic carbon substrates (b). 3-HP bicycle, 3-hydroxypropionic acid cycle; rTCA, reduced tricarboxylic acid cycle; CBB, Calvin Benson Bassham cycle; WL, Wood Ljungdahl pathway; DC/4HB, dicarboxylic acid/4-hydroxybutyrate cycle; 3HP/4HB, 3-hydroxypropionic acid/4-hydroxybutyrate cycle. CK: control treatment; W: warming treatment; N: nitrogen addition treatment; WN: warming nitrogen addition treatment.

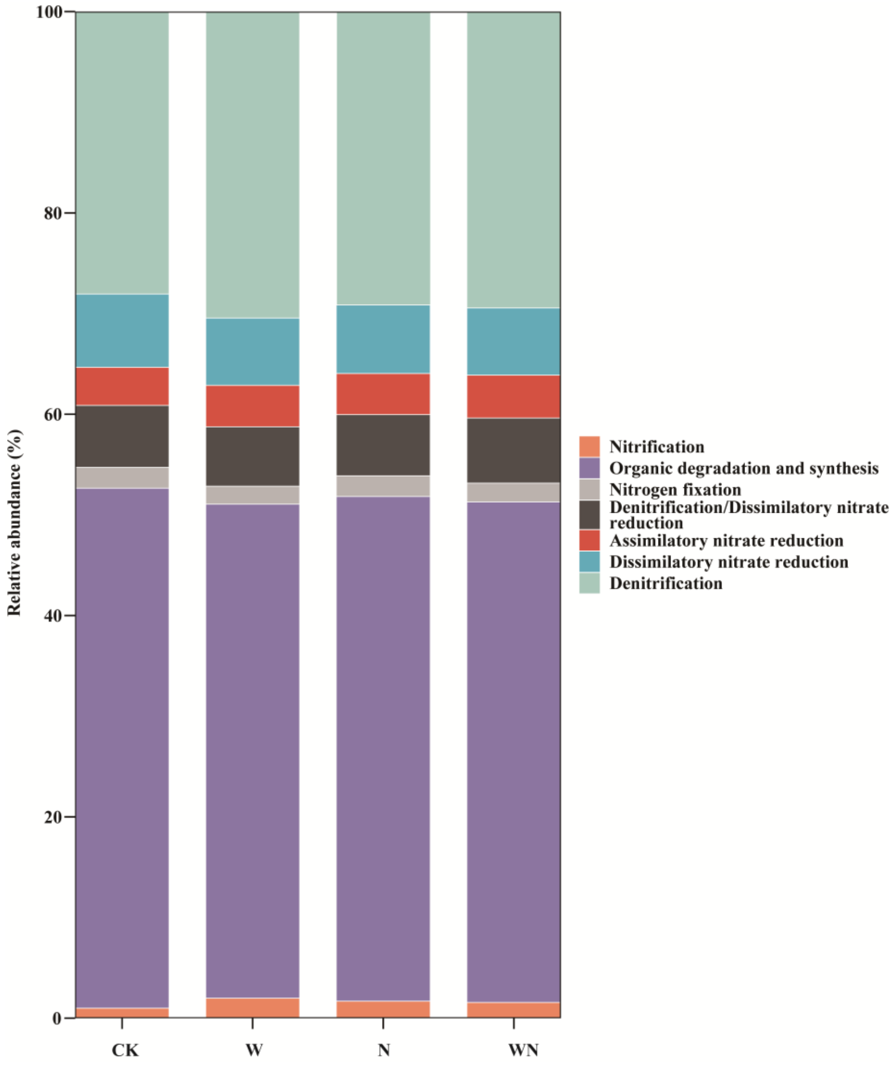

Figure S9: Effects of warming and nitrogen addition on the relative composition of soil microbial nitrogen cycle functional genes. CK: control treatment; W: warming treatment; N: nitrogen addition treatment; WN: warming nitrogen addition treatment.

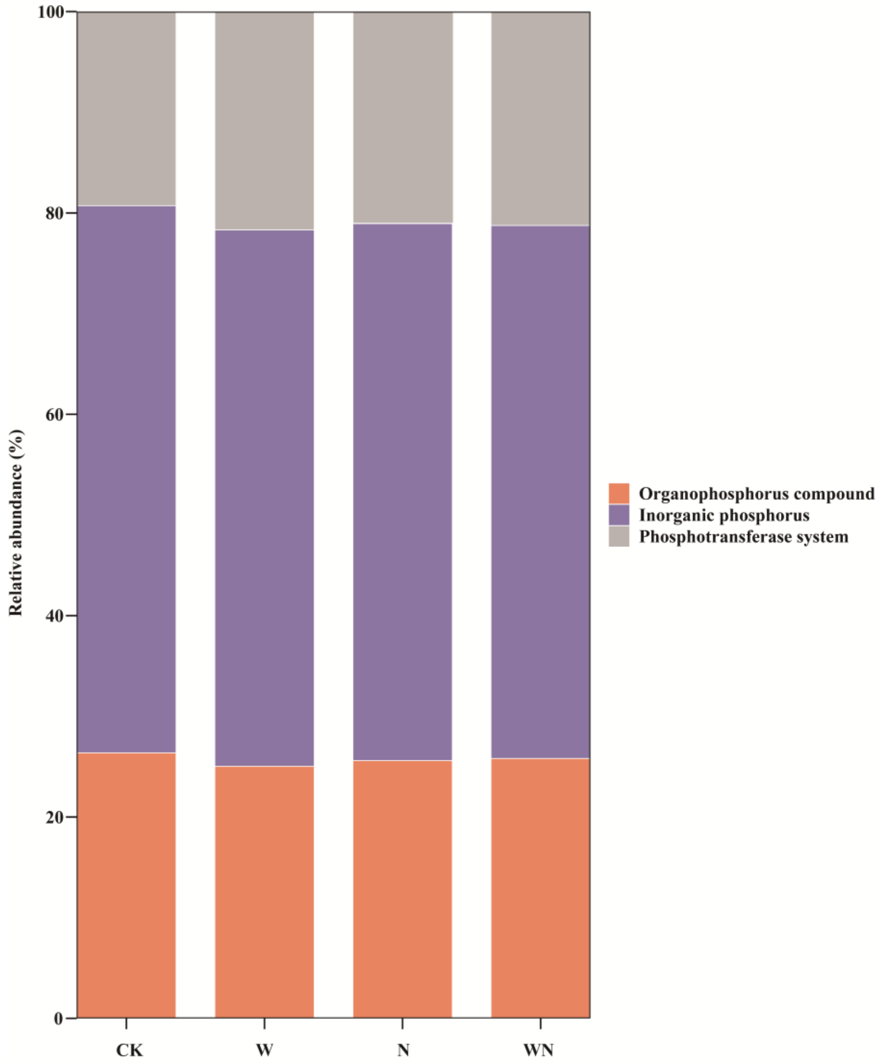

Figure S10: Effects of warming and nitrogen addition on the relative composition of soil microbial phosphorus cycle functional genes. CK: control treatment; W: warming treatment; N: nitrogen addition treatment; WN: warming nitrogen addition treatment.
